# Inhibitory Effects of Compounds and Extracts from* Ampelopsis brevipedunculata* on IL-6-Induced STAT3 Activation

**DOI:** 10.1155/2018/3684845

**Published:** 2018-06-10

**Authors:** Hyun-Jae Jang, Seung-Jae Lee, Hyung-Jin Lim, Kyungsook Jung, Soyoung Lee, Chan Sun Park, Seung Woong Lee, Mun-Chual Rho

**Affiliations:** Immunoregulatory Material Research Center, Korea Research Institute of Bioscience and Biotechnology, 181 Ipsin-gil, Jeongeup-si, Jeonbuk 56212, Republic of Korea

## Abstract

*Ampelopsis brevipedunculata* (Maxim.) Trautv. (AB), a traditional East Asian medicine, exhibits protective effects against several inflammatory diseases. Our search for an inhibitor of IL-6-induced JAK2/STAT3 activation revealed that AB ethanolic extract (ABE) had a significant inhibitory effect on IL-6-induced STAT3 expression in Hep3B cells. The isolation and purification of an EtOAc-soluble fraction of ABE (ABEA) using reversed-phase high-performance liquid chromatography (RP-HPLC) afforded 17 compounds. The structures of these compounds (**1**-**17**) were elucidated based on ^1^H and ^13^C nuclear magnetic resonance (NMR) spectroscopy as well as electrospray-ionization mass spectrometry (ESI-MS) data. ABE and ABEA were screened by a luciferase assay using Hep3B cells transfected with the STAT3 reporter gene. ABEA exhibited potent inhibitory effects on IL-6-induced STAT3 expression; moreover, these effects arose from the inhibition of the phosphorylation of the STAT3, JAK2, and ERK proteins in U266 cells. In addition, the compounds isolated from ABEA were measured for their inhibitory effects on IL-6-stimulated STAT3 expression. Of the compounds isolated, betulin showed the greatest inhibitory effects on IL-6-induced STAT3 activation in the luciferase assay (IC_50_ value: 3.12 *μ*M). Because of its potential for inhibiting STAT3 activation,* A. brevipedunculata* could be considered a source of compounds of pharmaceutical interest.

## 1. Introduction

Interleukin 6 (IL-6), which is involved in various physiological functions including neurodevelopment, haematopoiesis, bone metabolism, and immunity [1–4], is implicated in many inflammatory diseases, such as asthma, arthritis, osteoporosis, diabetes, cancer, ulcerative colitis, and Crohn's disease, and plays an important role in inflammation and immune diseases [5–7]. Thus, after the binding of IL-6 to its receptors, such as membrane-bound or soluble receptors (IL-6R or sIL-6R), the IL-6 complex induces the dimerization and phosphorylation of glycoprotein 130 (gp130). Activation of the IL-6 complex leads to a signalling cascade involving the Janus kinases (JAK)/signal transducer and activator of transcription 3 (STAT3). STAT3 is a member of the STAT family of proteins, and STAT3 plays a role in the response to growth factors and cytokines such as IL-6 [8]. STAT3 is phosphorylated by receptor-associated JAK2 to form homo- or heterodimers and then translocates to the nucleus where it acts as a transcription activator [9]. JAK2 provides “instructions” for producing proteins that promote the growth, development, and proliferation of cells and mediates essential signalling events involved in both innate and adaptive immunity. JAK2/STAT activation has been observed in several types of cancer and tumours, including multiple myeloma, leukaemia, prostate cancer, breast cancer, and colon cancer [10, 11]. The JAK2/STAT signalling pathways is thus very important for cell growth and differentiation, and natural products that target JAK2/STAT have potential for the treatment of cancer and tumours [11].

*Ampelopsis brevipedunculata* (Maxim.) Trautv. (AB) has beneficial effects on human health, including antioxidant, antihypertension, antiviral, antimutagenic, and skin and liver (antihepatotoxic) protective activities [12, 13]. In Asia, AB has been used in traditional medicine to treat several diseases. Although the components of AB, such as oligostilbenes [14] and phenolics [15], have been reported, the phytochemical constituents of AB are not well known. Therefore, the development of new technologies to identify the bioactive compounds in AB will increase the value of this medicinal plant and will present unique challenges and opportunities for further study.

Thus, the objectives of the present study were to (1) measure the inhibitory effects of ABE and ABEA on Il-6-induced STAT3 activity, (2) evaluate the STAT3, JAK2, and ERK protein levels in the ABEA fraction, (3) and isolate and identify the constituents of ABEA using RP-HPLC, NMR, and ESI-MS. This study will provide a better understanding of the relationship between IL-6 activity and the extracts and compounds.

## 2. Materials and Methods

### 2.1. General Procedures

^1^H, ^13^C, and 2D NMR spectroscopic data were recorded on a JEOL JNM-ECA600 or JEOL JNM-EX400 instrument (JEOL, Tokyo, Japan) using TMS as a reference. Column chromatography was performed with silica gel (Kieselgel 60, 230-400 mesh, Merck, Darmstadt, Germany), and silica gel 60 F_254_ and RP-18 F_254_s (Merck) were used for TLC profiling. Medium-pressure liquid chromatography (MPLC) was performed using a Combiflash RF (Teledyne Isco, Lincoln, NE, USA), and preparative HPLC was performed on a Shimadzu LC-6AD (Shimadzu Co., Tokyo, Japan) instrument equipped with an SPD-20A detector using a Phenomenex Luna C_18_ (250 mm × 21.2 mm, 5 *μ*m, Phenomenex, Torrance, CA, USA) and YMC C_18_ hydrosphere (250 mm × 20 mm, S-5 *μ*m, YMC Co., Kyoto, Japan).

### 2.2. Plant Material

AB was purchased from an herbal store in Seoul, Korea, in May 2014. One of the authors (M. -C. Rho) performed the botanical identification, and a voucher specimen (KRIBB-KR2013-1) has been deposited at the laboratory of the Immunoregulatory Material Research Center at the Jeonbuk Branch of the Korea Research Institute of Bioscience and Biotechnology.

### 2.3. Extraction and Isolation

Dried and pulverized parts of AB vines (9 kg) were extracted at room temperature with 95% EtOH, and the combined extract was evaporated* in vacuo* to obtain the EtOH extract (337.8 g). The EtOH extract was suspended in H_2_O (1.5 L) and progressively partitioned with EtOAc (ABE, 130.1 g). ABEA was separated into 38 subfractions (ABE1-ABE38) by silica gel column chromatography with a solvent system comprising a stepwise gradient of CHCl_3_/MeOH (1:0–0:1, v/v). ABE3 (187.9 mg) was found to contain *β*-sitosterol (**3**), and ABE7 was purified by recrystallization from MeOH to afford** 4** (105.7 mg). ABE19 (16.7 g) was subjected to silica gel column chromatography using a stepwise solvent system composed of CHCl_3_ and MeOH (50:1–0:1, v/v) and separated into 13 subfractions (ABE19A–ABE19M). ABE19B (32.1 mg) was rechromatographed by MPLC (silica gel, 40 g, CHCl_3_/MeOH, 50:1–0:1, v/v) to yield** 1** (2.6 mg), and ABE19D (112.0 mg) was separated by MPLC (silica gel, 40 g, hexane/EtOAc, 1:0–0:1, v/v) to yield** 2** (3.5 mg). ABE19H (2.9 g) was further purified by MPLC (C_18_ 130 g, 10–100% MeOH), and** 16 **(4.3 mg) was separated from ABE19H9 by recrystallization. ABE23 (10.8 g) was chromatographed on an MPLC silica column (silica gel 120 g,* n*-hexane/EtOAc, 5:1–0:1, v/v) to yield 13 subfractions (ABE23A–ABE23M), and ABE23E (335.2 mg) and ABE23L (56.1 mg) were found to contain compounds** 10** and** 17**, respectively. ABE23A (767.1 mg) was purified by MPLC (silica gel, 40 g, CHCl_3_/MeOH, 50:1–0:1, v/v) and afforded 6 subfractions (ABE23A1–ABE23A6). Compounds** 9** (3.1 mg) and** 12** (4.3 mg) were isolated from ABE23A4 (283.4 mg) using preparative HPLC (C_18_, 15% MeOH, 6 mL/min). Compound** 5** (157.4 mg) was isolated from ABE23G (875.5 mg) using MPLC (silica gel, 40 g,* n*-Hexane/EtOAc, 1:0–0:1, v/v). Compound** 8** was obtained from ABE25 (2.0 g) using MPLC (silica gel 120 g, CHCl_3_:MeOH, 1:0–7:3, v/v), and ABE27 (37.6 g) was chromatographed on a silica gel column eluted with CHCl_3_ and MeOH (1:0–0:1, v/v) to yield** 7** (4.5 g). ABE28 (28.1 g) was subjected to silica gel column chromatography (CHCl_3_/MeOH, 1:0–0:1, v/v). Among the resulting subfractions (ABE28A–ABE28H), ABE28E (3.5 g) was separated by MPLC (C_18_ 130 g, 10–100% MeOH) to yield 9 fractions (ABE28E1–ABE28E9); compound** 15** (4.6 mg) was obtained by the recrystallization of ABE28E7. ABE28E2 (537.2 mg) was separated by MPLC (C_18_ 130 g, 10–100% MeOH) to yield 13 subfractions (ABE28E2A–ABE28E2M). Preparative HPLC (YMC C_18_ hydrosphere, 10–20% CH_3_CN, 6 mL/min) was used to isolate compounds** 11** (1.5 mg),** 6** (5.3 mg), and** 13** (14.4 mg) from ABE28E2C (63.2 mg), ABE28E2G (37.7 mg), and ABE28E2H (59.2 mg), respectively. ABE32 (8.6 g) was subjected to silica gel column chromatography, eluted with a mixture of CHCl_3_ and MeOH (1:0–0:1, v/v), and yielded 10 subfractions (ABE32A–ABE32J). Compound** 14** (3.5 mg) was isolated by recrystallization of ABE32I (159.8 mg).

### 2.4. Cell Culture and Viability

Hep3B (ATCC HB-8064) and U266 (ATCC TIB-196™) cells were maintained in Dulbecco's modified Eagle's medium (DMEM) and RPMI 1640 medium supplemented with 10% foetal bovine serum, 2 mM glutamine, 100 U/mL penicillin, and 100 mg/mL streptomycin sulfate. Cells were maintained in exponential growth phase at 37°C in humidified air with 5% CO_2_. The 3-(4,5-dimethylthiazol-2-yl)-2,5-diphenyltetrazolium bromide (MTT) assay was performed to assess cell viability [16]. Cells were added to each well of a 96-well microplate and incubated at 37°C for 24 h. The cytotoxicity of each fraction was screened at concentrations of 1, 5, 10, and 50 *μ*g/mL. After treatment with the EtOAc or water fraction for 24 h, MTT solution was added to each well, and the plate was incubated for 2 h. To stop succinate-tetrazolium reductase activity and solubilize formazan crystals, 200 *μ*L of dimethyl sulfoxide (DMSO) was added to each well and incubated at 37°C for 1 h. The absorbance of each well was measured at 563 nm using an ELISA reader (BioRad, Model 680, Hercules, CA, USA). Cell viability was expressed as a percentage of the cell viability of the control cells.

### 2.5. pSTAT3 Luciferase Assay

Human hepatoma Hep3B cells (ATCC HB-8064) stably expressing pSTAT3-Luc were established as described previously [17]. In brief, Hep3B cells expressing pSTAT3-luciferase were seeded in a 96-well culture plate at 2 × 10^4^ cells/well, and the cells were treated with samples for 1 h before stimulation with IL-6 (10 ng/mL) for 12 h. Luciferase activity was measured according to the manufacturer's protocol (Promega Corp., Madison, WI, USA).

### 2.6. Western Blotting

Cell proteins were separated by centrifuging the cell lysate for 20 min at 13,000 ×* g*. Equivalent amounts of protein were separated by 10% sodium dodecyl sulfate-polyacrylamide gels (SDS-PAGE) and transferred to polyvinylidene fluoride (PVDF) membranes using an electroblotting apparatus. The blots were then immersed in freshly prepared 5% skim milk for a blocking step at room temperature (1 h). Next, the membranes were incubated overnight at 4°C with primary antibody, including anti-phospho-STAT3 (1:1000), anti-STAT3 (1:1000), anti-phospho-JAK2 (1:1000), anti-JAK2 (1:1000), anti-phospho-ERK (1:1000), and anti-ERK (1:1000) antibodies (Cell Signaling, Beverly, MA, USA). The membranes were then incubated with an appropriate horseradish peroxide-conjugated secondary antibody (1:5000) at room temperature. The optical densities of the antibody-specific bands were analysed using a Luminescent Image Analyzer, LAS-3000 (Fuji, Tokyo, Japan) [18].

### 2.7. Statistical Analysis

All experimental data were obtained in triplicate assays. Data are expressed as the means ± standard deviation (SD). Statistical analyses were performed using Student's* t*-test for paired data. Statistical analyses were performed using Prism 5 software (GraphPad software, San Diego, CA, USA). Student's* t*-test was used to evaluate the data, and differences were considered statistically significant at* P* < 0.05 for the mean values of triplicate samples.

## 3. Results

### 3.1. Preparation of Extracts and Determination of STAT3 Inhibition

**Figure 1:**
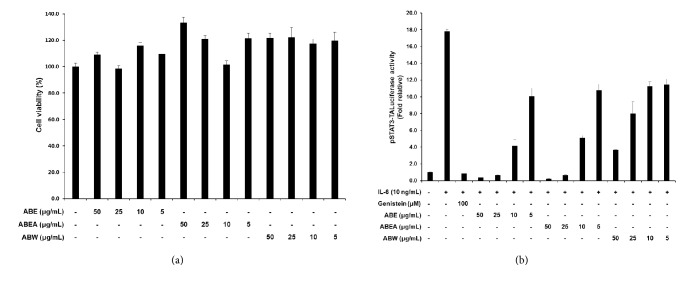
The viability of Hep3B cells after treatment with the ethanol (ABE), ethyl acetate (ABEA), and water fractions (ABW) obtained from* A. brevipedunculata* (a). The inhibitory effects of ABE, ABEA, and ABW on IL-6/STAT3 activation in Hep3B cells were determined using a luciferase assay (b). Genistein was used as a positive control. Values are the means ± SE; ^*∗*^*p* < 0.05, compared with the IL-6-treated group.

To examine the cytotoxic effects of ABE, ABEA, and water-soluble fractions from ABE (ABW) against Hep3B cells, MTT assays were performed. Cells were treated with various concentrations of these fractions (0, 5, 10, 25, and 50 *μ*g/mL) for 24 h. The cell viability was not affected by ABE, ABEA, or ABW at 50 *μ*g/mL (Figure 1), but at this concentration, the extracts and fractions significantly inhibited IL-6-induced STAT3 expression in Hep3B cells. Notably, ABE and ABEA exhibited stronger inhibitory effects than ABW in a luciferase assay (Figure 1). These data demonstrated that the nonpolar components in ABEA are more potent inhibitors of IL-6-induced STAT3 activity than the polar components of ABW.

### 3.2. Inhibitory Effects of ABEA on IL-6-Induced JAK2/STAT3 Phosphorylation

**Figure 2:**
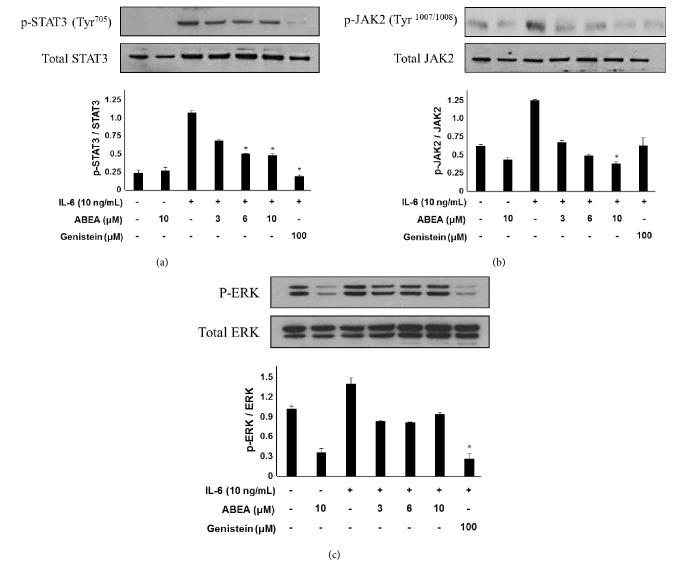
Inhibitory effects of JAK2, STAT3, and ERK activation by ABEA on IL-6-stimulated U266 cell lines. Cells were preincubated with ABEA for 1 h before stimulation with IL-6 (10 ng/mL). Western blot analysis indicated that ABEA inhibited the phosphorylation of JAK2, STAT3, and ERK in U266 cells. The total amount of the corresponding nonphosphorylated protein was used as a loading control for the phosphorylated proteins. Genistein was used as a positive control. Values are the means ± SE; ^*∗*^*p* < 0.05 compared with the IL-6-treated group.

To characterize the molecular mechanisms by which ABEA inhibits JAK2, STAT3, and ERK activity, we examined various transcription-related proteins. Western blot analysis demonstrated that the IL-6-induced phosphorylation of STAT3 and JAK2 was reduced in U266 cells after pretreatment with ABEA at 3, 6, and 10 *μ*g/mL (Figures 2(a) and 2(b)). Although a reduction in the pERK/ERK ratio was observed after treatment with ABEA, no significant dose-dependent differences were observed compared to JAK2/STAT3 activation in IL-6-induced U266 cells (Figure 2(c)).

### 3.3. Isolation of Active Compounds

**Figure 3:**
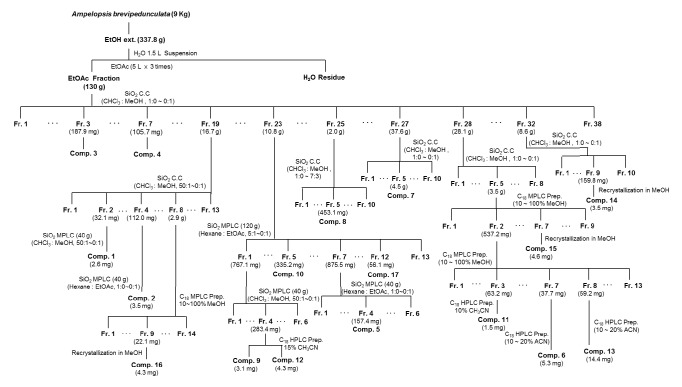
Schematic diagram for the isolation of compounds** 1**-**17** from* A. brevipedunculata*.

**Figure 4:**
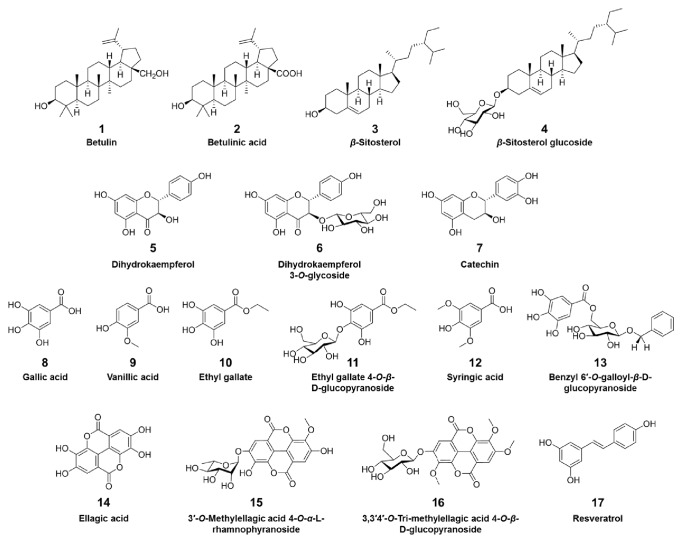
Chemical structures of compounds** 1**-**17** from* A. brevipedunculata*.

Bioactivity-guided fractionation and column chromatography purification (Figure 3) of ABEA led to the isolation of compounds** 1**-**17** (Figure 4). The compounds were identified as betulin (**1**) [19], betulinic acid (**2**) [20], *β*-sitosterol (**3**) [21], *β*-sitosterol glucoside (**4**) [22], dihydrokaempferol (**5**) [23], dihydrokaempferol 3-*O*-glycoside (**6**) [24], catechin (**7**) [25], gallic acid (**8**) [26], vanillic acid (**9**) [27], ethyl gallate (**10**) [28], ethyl gallate 4-*O*-*β*-D-glucopyranoside (**11**) [29], syringic acid (**12**) [30], benzyl 6′-*O*-galloyl-*β*-D-glucopyranoside (**13**) [31], ellagic acid (**14**) [32], 3′-*O*-methylellagic acid 4-*O*-*α*-L-rhamnopyranoside** (15**) [33], 3,3′4′-O-tri-methylellagic acid 4-*O*-*β*-D-glucopyranoside (**16**) [32], and resveratrol (**17**) [34] by comparison with the spectroscopic data in the literature (Supplementary Material, Figure S1-51). Compounds** 3**,** 4**,** 6**,** 11**,** 13**, and** 14**-**16** were isolated from this plant for the first time, and compounds** 1**,** 2**,** 5**,** 8**,** 10-14**, and** 17** exhibited half-maximal inhibitory concentration (IC_50_) values ranging from 3.1 to 49.7 *μ*M against IL-6-induced STAT3 activation (Table 1); additionally, with the exception of betulinic acid (**2**), these compounds showed no cytotoxicity at their IC_50_ concentrations (Supplementary Material, Figure S52). Betulin (**1**) exhibited the greatest inhibitory effects on IL-6-induced STAT3 activation with an IC_50_ value of 3.12 *μ*M.

## 4. Discussion

AB has been reported to have various pharmacological activities, such as skin and liver protection and antioxidant activities [12, 35]. In 2014, the anti-inflammatory activity of ABW via inhibition of the PKC-mediated JNK/NF-*κ*B signalling pathways was reported [36]; however, the inhibitory activity of ABEA on IL-6-mediated diseases, such as inflammation, cancer cachexia, rheumatoid arthritis, hypercalcaemia, and multiple myeloma [37], through JAK2/STAT3 signal transduction has not been reported.

IL-6 is a multifunctional cytokine that plays major roles in host defence and immune reactions [38]. Increased levels of IL-6 have been reported in various pathological conditions, such as infection (bacteria and virus), immune disease, inflammation, and tumours [39]. Therefore, regulation of IL-6 function might be effective against various diseases. IL-6 binds to homo- or heterodimer membrane receptor complexes containing glycoprotein 130 (gp130) or the leukaemia inhibitory factor receptor (LIFR) and activates the JAK family of tyrosine kinases [40]. In particular, phosphorylated JAK2 recruits Grb2/Shc/Ras/MEK1/2 and then activates ERK1/2 independently of SHP2 [41]. In Figure 2(c), ABEA suppresses IL-6-mediated ERK phosphorylation; however, no significant differences were observed for at concentrations ranging from 3 to 10 *μ*M compared to the JAK2/STAT3 inhibitory activation. Accordingly, significant differences in the ERK inhibitory activity of ABEA can be observed at concentrations higher than 10 *μ*M, and the main signalling transduction of the IL-6 cascade pathway affected by ABEA is the JAK2/STAT3 signalling pathway.

Except for some phenolics and triterpenes, the chemical constituents of AB are not well known, and our results may provide useful information on the phytochemical properties of AB. We investigated the components of AB using repeated column chromatography. Among the compounds isolated from ABEA,** 1**,** 2**,** 5**,** 8**,** 10**-**14**, and** 17** exert inhibitory effects on IL-6-stimulated STAT3 expression, and betulin (**1**) exhibited the greatest inhibitory activity (Table 1). Betulin, a lupane-type pentacyclic triterpenoid, has various biological activities, such as anti-HIV, anticancer, and anti-inflammatory effects [42, 43], and is a major component in the bark of birch trees such as* Betula pendula, B. papyrifera*, and* B. neoalaskana*, which were traditionally used to treat eczema, psoriasis, inflammation, rheumatism, and arthritic diseases [44–47].

Based on the present data, we isolated and identified 17 compounds from ABEA, and those compounds were tested for their ability to inhibit IL-6/STAT3 activation using a luciferase reporter assay. In addition, the ABEA fraction was presumed to be involved in inhibiting the expression of genes such as those for STAT3, JAK2, and ERK, which are involved in various signalling pathways. Consequently, the extracts and constituents of AB displayed potent natural inhibition of IL-6/STAT3 in an* in vitro* model. Overall, the extracts and components have the potential to be developed into new biomaterials. However, further studies are needed to elucidate the precise mechanism of* in vivo* activity.

## Figures and Tables

**Table 1:** Inhibitory effects of the major compounds from* Ampelopsis brevipedunculata* on IL-6/STAT3 activation.

Compound	IC_50_ (*μ*M)	Compound	IC_50_ (*μ*M)
Betulin (**1**)	3.12 ± 0.31	Ethyl gallate (**10**)	44.13 ± 3.16
Betulinic acid (**2**)	6.98 ± 0.34	Ethyl gallyl 4-O-*α*-D-glucopyranoside (**11**)	22.85 ± 0.74
*β*-Sitosterol (**3**)	> 50	Syringic acid (**12**)	15.82 ± 4.70
*β*-Sitosterol glucoside (**4**)	> 50	Benzyl 6′-O-galloyl-*β*-D-glucopyranoside (**13**)	4.99 ± 2.02
Aromadendrin (**5**)	49.73 ± 10.15	Ellagic acid (**14**)	4.85 ± 1.73
3-O-Glycoside-aromadendrin (**6**)	> 50	3′-O-Methylellagic acid 4-O-*α*-L-rhamnopyranoside** (15**)	> 50
Catechin (**7**)	> 50	4′-O-(*β*-D-Glucopyranosyl)-3,3′4-tri-O-methylellagic acid (**16**)	> 50
Gallic acid (**8**)	27.30 ± 3.58	Resveratrol (**17**)	21.18 ± 1.10
Vanillic acid (**9**)	> 50	Genistein^a^	13.60 ± 0.36

^a^ Genistein was used as the positive control.

## Supplementary Materials

Supplementary Materials Figure S1: ESI-MS spectrum of** 1**. Figure S2: ^1^H NMR (400 MHz, chloroform-*d*) spectrum of** 1**. Figure S3: ^13^C NMR (100 MHz, chloroform-*d*) spectrum of** 1**. Figure S4: ESI-MS spectrum of** 2**. Figure S5: ^1^H NMR (600 MHz, pyridine-*d*_5_) spectrum of** 2**. Figure S6: ^13^C NMR (150 MHz, pyridine-*d*_5_) spectrum of** 2**. Figure S7: GC-MS spectrum of** 3**. Figure S8: ^1^H NMR (400 MHz, chloroform-*d*) spectrum of** 3**. Figure S9: ^13^C NMR (100 MHz, chloroform-*d*) spectrum of** 3**. Figure S10: FABMS spectrum of** 4**. Figure S11: ^1^H NMR (600 MHz, pyridine-*d*_5_) spectrum of** 4**. Figure S12: ^13^C NMR (150 MHz, pyridine-*d*_5_) spectrum of** 4**. Figure S13: ESI-MS spectrum of** 5**. Figure S14: ^1^H NMR (400 MHz, methanol-*d*_4_) spectrum of** 5**. Figure S15: ^13^C NMR (100 MHz, methanol-*d*_4_) spectrum of** 5**. Figure S16: ESI-MS spectrum of** 6**. Figure S17: ^1^H NMR (400 MHz, methanol-*d*_4_) spectrum of** 6**. Figure S18: ^13^C NMR (100 MHz, methanol-*d*_4_) spectrum of** 6**. Figure S19: ESI-MS spectrum of** 7**. Figure S20: ^1^H NMR (400 MHz, methanol-*d*_4_) spectrum of** 7**. Figure S21: ^13^C NMR (100 MHz, methanol-*d*_4_) spectrum of** 7**. Figure S22: ESI-MS spectrum of** 8**. Figure S23: ^1^H NMR (400 MHz, methanol-*d*_4_) spectrum of** 8**. Figure S24: ^13^C NMR (100 MHz, methanol-*d*_4_) spectrum of** 8**. Figure S25: ESI-MS spectrum of** 9**. Figure S26: ^1^H NMR (600 MHz, methanol-*d*_4_) spectrum of** 9**. Figure S27: ^13^C NMR (150 MHz, methanol-*d*_4_) spectrum of** 9**. Figure S28: ESI-MS spectrum of** 10**. Figure S29: ^1^H NMR (400 MHz, methanol-*d*_4_) spectrum of** 10**. Figure S30: ^13^C NMR (100 MHz, methanol-*d*_4_) spectrum of** 10**. Figure S31: ESI-MS spectrum of** 11**. Figure S32: ^1^H NMR (400 MHz, methanol-*d*_4_) spectrum of** 11**. Figure S33: ^13^C NMR (100 MHz, methanol-*d*_4_) spectrum of** 11**. Figure S34: ESI-MS spectrum of** 12**. Figure S35: ^1^H NMR (600 MHz, methanol-*d*_4_) spectrum of** 12**. Figure S36: ^13^C NMR (150 MHz, methanol-*d*_4_) spectrum of** 12**. Figure S37: ESI-MS spectrum of** 13**. Figure S38: ^1^H NMR (400 MHz, methanol-*d*_4_) spectrum of** 13**. Figure S39: ^13^C NMR (100 MHz, methanol-*d*_4_) spectrum of** 13**. Figure S40: ESI-MS spectrum of** 14**. Figure S41: ^1^H NMR (600 MHz, DMSO-*d*_6_) spectrum of** 14**. Figure S42: ^13^C NMR (100 MHz, DMSO-*d*_6_) spectrum of** 14**. Figure S43: ESI-MS spectrum of** 15**. Figure S44: ^1^H NMR (600 MHz, DMSO-*d*_6_) spectrum of** 15**. Figure S45: ^13^C NMR (150 MHz, DMSO-*d*_6_) spectrum of** 15**. Figure S46: ESI-MS spectrum of** 16**. Figure S47: ^1^H NMR (600 MHz, DMSO-*d*_6_) spectrum of** 16**. Figure S48: ^13^C NMR (150 MHz, DMSO-*d*_6_) spectrum of** 16**. Figure S49: ESI-MS spectrum of** 17**. Figure S50: ^1^H NMR (400 MHz, methanol-*d*_4_) spectrum of** 17**. Figure S51: ^13^C NMR (100 MHz, methanol-*d*_4_) spectrum of** 17**. Figure S52: Cytotoxicity of compounds 1-17 on Hep3B-STAT3-Luc cell lines.
