# Supplementary material for: Inhibitory Effects of Compounds and Extracts from Ampelopsis brevipedunculata on IL-6-Induced STAT3 Activation
Source: Biomed Res Int. 2018 Jun 10;2018:3684845. doi: 10.1155/2018/3684845 (PMC6015723; doi:10.1155/2018/3684845)
Supplement: Supplementary Materials — Figure S1: ESI-MS spectrum of 1. Figure S2: 1H NMR (400 MHz, chloroform-d) spectrum of 1. Figure S3: 13C NMR (100 MHz, chloroform-d) spectrum of 1. Figure S4: ESI-MS spectrum of 2. Figure S5: 1H NMR (600 MHz, pyridine-d5) spectrum of 2. Figure S6: 13C NMR (150 MHz, pyridine-d5) spectrum of 2. Figure S7: GC-MS spectrum of 3. Figure S8: 1H NMR (400 MHz, chloroform-d) spectrum of 3. Figure S9: 13C NMR (100 MHz, chloroform-d) spectrum of 3. Figure S10: FABMS spectrum of 4. Figure S11: 1H NMR (600 MHz, pyridine-d5) spectrum of 4. Figure S12: 13C NMR (150 MHz, pyridine-d5) spectrum of 4. Figure S13: ESI-MS spectrum of 5. Figure S14: 1H NMR (400 MHz, methanol-d4) spectrum of 5. Figure S15: 13C NMR (100 MHz, methanol-d4) spectrum of 5. Figure S16: ESI-MS spectrum of 6. Figure S17: 1H NMR (400 MHz, methanol-d4) spectrum of 6. Figure S18: 13C NMR (100 MHz, methanol-d4) spectrum of 6. Figure S19: ESI-MS spectrum of 7. Figure S20: 1H NMR (400 MHz, methanol-d4) spectrum of 7. Figure S21: 13C NMR (100 MHz, methanol-d4) spectrum of 7. Figure S22: ESI-MS spectrum of 8. Figure S23: 1H NMR (400 MHz, methanol-d4) spectrum of 8. Figure S24: 13C NMR (100 MHz, methanol-d4) spectrum of 8. Figure S25: ESI-MS spectrum of 9. Figure S26: 1H NMR (600 MHz, methanol-d4) spectrum of 9. Figure S27: 13C NMR (150 MHz, methanol-d4) spectrum of 9. Figure S28: ESI-MS spectrum of 10. Figure S29: 1H NMR (400 MHz, methanol-d4) spectrum of 10. Figure S30: 13C NMR (100 MHz, methanol-d4) spectrum of 10. Figure S31: ESI-MS spectrum of 11. Figure S32: 1H NMR (400 MHz, methanol-d4) spectrum of 11. Figure S33: 13C NMR (100 MHz, methanol-d4) spectrum of 11. Figure S34: ESI-MS spectrum of 12. Figure S35: 1H NMR (600 MHz, methanol-d4) spectrum of 12. Figure S36: 13C NMR (150 MHz, methanol-d4) spectrum of 12. Figure S37: ESI-MS spectrum of 13. Figure S38: 1H NMR (400 MHz, methanol-d4) spectrum of 13. Figure S39: 13C NMR (100 MHz, methanol-d4) spectrum of 13. Figure S40: ESI-MS spectrum of 14. Figure S41: 1H NMR (600 [file 3684845.f1.docx]

**Supplementary Material**

**Inhibitory effects of compounds and extracts from *Ampelopsis brevipedunculata* on IL-6-induced STAT3 activation**

Hyun-Jae Jang^†^, Seung-Jae Lee^†^, Hyung-Jin Lim, Kyungsook Jung, Soyoung Lee, Chan Sun Park, Seung Woong Lee*, and Mun-Chual Rho*

Immunoregulatory Material Research Center, Korea Research Institute of Bioscience and Biotechnology, 181 Ipsin-gil, Jeongeup-si, Jeonbuk 56212, Korea

^†^ Hyun-Jae Jang and Seung-Jae Lee equally contributed to this study.

**Corresponding Authors**

*(S. Lee) Tel: 82-63-570-5264. Fax: 82-63-570-5239. E-mail: [lswdoc@kribb.re.kr](mailto:lswdoc@kribb.re.kr)

*(M. Rho) Tel: 82-63-570-5230. Fax: 82-63-570-5239. E-mail: [rho-m@kribb.re.kr](mailto:rho-m@kribb.re.kr)

| **Content** | | **Page** |
| --- | --- | --- |
| **Figure S1.** | ESIMS spectrum of **1** | S1 |
| **Figure S2.** | ^1^H NMR (400 MHz, chloroform-*d*) spectrum of **1** | S2 |
| **Figure S3** | ^13^C NMR (100 MHz, chloroform-*d*) spectrum of **1** | S3 |
| **Figure S4** | ESIMS spectrum of **2** | S4 |
| **Figure S5** | ^1^H NMR (600 MHz, pyridine-*d*_5_) spectrum of **2** | S5 |
| **Figure S6** | ^13^C NMR (150 MHz, pyridine-*d*_5_) spectrum of **2** | S6 |
| **Figure S7** | GC-MS spectrum of **3** | S7 |
| **Figure S8** | ^1^H NMR (400 MHz, chloroform-*d*) spectrum of **3** | S8 |
| **Figure S9.** | ^13^C NMR (100 MHz, chloroform-*d*) spectrum of **3** | S9 |
| **Figure S10.** | FABMS spectrum of **4** | S10 |
| **Figure S11.** | ^1^H NMR (600 MHz, pyridine-*d*_5_) spectrum of **4** | S11 |
| **Figure S12.** | ^13^C NMR (150 MHz, pyridine-*d*_5_) spectrum of **4** | S12 |
| **Figure S13.** | ESIMS spectrum of **5** | S13 |
| **Figure S14.** | ^1^H NMR (400 MHz, methanol-*d*_4_) spectrum of **5** | S14 |
| **Figure S15.** | ^13^C NMR (100 MHz, methanol-*d*_4_) spectrum of **5** | S15 |
| **Figure S16.** | ESIMS spectrum of **6** | S16 |
| **Figure S17.** | ^1^H NMR (400 MHz, methanol-*d*_4_) spectrum of **6** | S17 |
| **Figure S18.** | ^13^C NMR (100 MHz, methanol-*d*_4_) spectrum of **6** | S18 |
| **Figure S19.** | ESIMS spectrum of **7** | S19 |
| **Figure S20.** | ^1^H NMR (400 MHz, methanol-*d*_4_) spectrum of **7** | S20 |
| **Figure S21.** | ^13^C NMR (100 MHz, methanol-*d*_4_) spectrum of **7** | S21 |
| **Figure S22.** | ESIMS spectrum of **8** | S22 |
| **Figure S23.** | ^1^H NMR (400 MHz, methanol-*d*_4_) spectrum of **8** | S23 |
| **Figure S24.** | ^13^C NMR (100 MHz, methanol-*d*_4_) spectrum of **8** | S24 |
| **Figure S25.** | ESIMS spectrum of **9** | S25 |
| **Figure S26.** | ^1^H NMR (600 MHz, methanol-*d*_4_) spectrum of **9** | S26 |
| **Figure S27.** | ^13^C NMR (150 MHz, methanol-*d*_4_) spectrum of **9** | S27 |
| **Figure S28.** | ESIMS spectrum of **10** | S28 |
| **Figure S29.** | ^1^H NMR (400 MHz, methanol-*d*_4_) spectrum of **10** | S29 |
| **Figure S30.** | ^13^C NMR (100 MHz, methanol-*d*_4_) spectrum of **10** | S30 |
| **Figure S31.** | ESIMS spectrum of **11** | S31 |
| **Figure S32.** | ^1^H NMR (400 MHz, methanol-*d*_4_) spectrum of **11** | S32 |
| **Figure S33.** | ^13^C NMR (100 MHz, methanol-*d*_4_) spectrum of **11** | S33 |
| **Figure S34.** | ESIMS spectrum of **12** | S34 |
| **Figure S35.** | ^1^H NMR (600 MHz, methanol-*d*_4_) spectrum of **12** | S35 |
| **Figure S36.** | ^13^C NMR (150 MHz, methanol-*d*_4_) spectrum of **12** | S36 |
| **Figure S37.** | ESIMS spectrum of **13** | S37 |
| **Figure S38.** | ^1^H NMR (400 MHz, methanol-*d*_4_) spectrum of **13** | S38 |
| **Figure S39.** | ^13^C NMR (100 MHz, methanol-*d*_4_) spectrum of **13** | S39 |
| **Figure S40.** | ESIMS spectrum of **14** | S40 |
| **Figure S41.** | ^1^H NMR (600 MHz, DMSO-*d*_6_) spectrum of **14** | S41 |
| **Figure S42.** | ^13^C NMR (100 MHz, DMSO-*d*_6_) spectrum of **14** | S42 |

| **Content** | | **Page** |
| --- | --- | --- |
| **Figure S43.** | ESIMS spectrum of **15** | S43 |
| **Figure S44.** | ^1^H NMR (600 MHz, DMSO-*d*_6_) spectrum of **15** | S44 |
| **Figure S45** | ^13^C NMR (150 MHz, DMSO-*d*_6_) spectrum of **15** | S45 |
| **Figure S46** | ESIMS spectrum of **16** | S46 |
| **Figure S47** | ^1^H NMR (600 MHz, DMSO-*d*_6_) spectrum of **16** | S47 |
| **Figure S48** | ^13^C NMR (150 MHz, DMSO-*d*_6_) spectrum of **16** | S48 |
| **Figure S49** | ESIMS spectrum of **17** | S49 |
| **Figure S50** | ^1^H NMR (400 MHz, methanol-*d*_4_) spectrum of **17** | S50 |
| **Figure S51.** | ^13^C NMR (100 MHz, methanol-*d*_4_) spectrum of **17** | S51 |
| **Figure S52.** | Cytotoxicity of compounds **1**-**17** on Hep3B-STAT3-Luc cell lines | S52 |

**
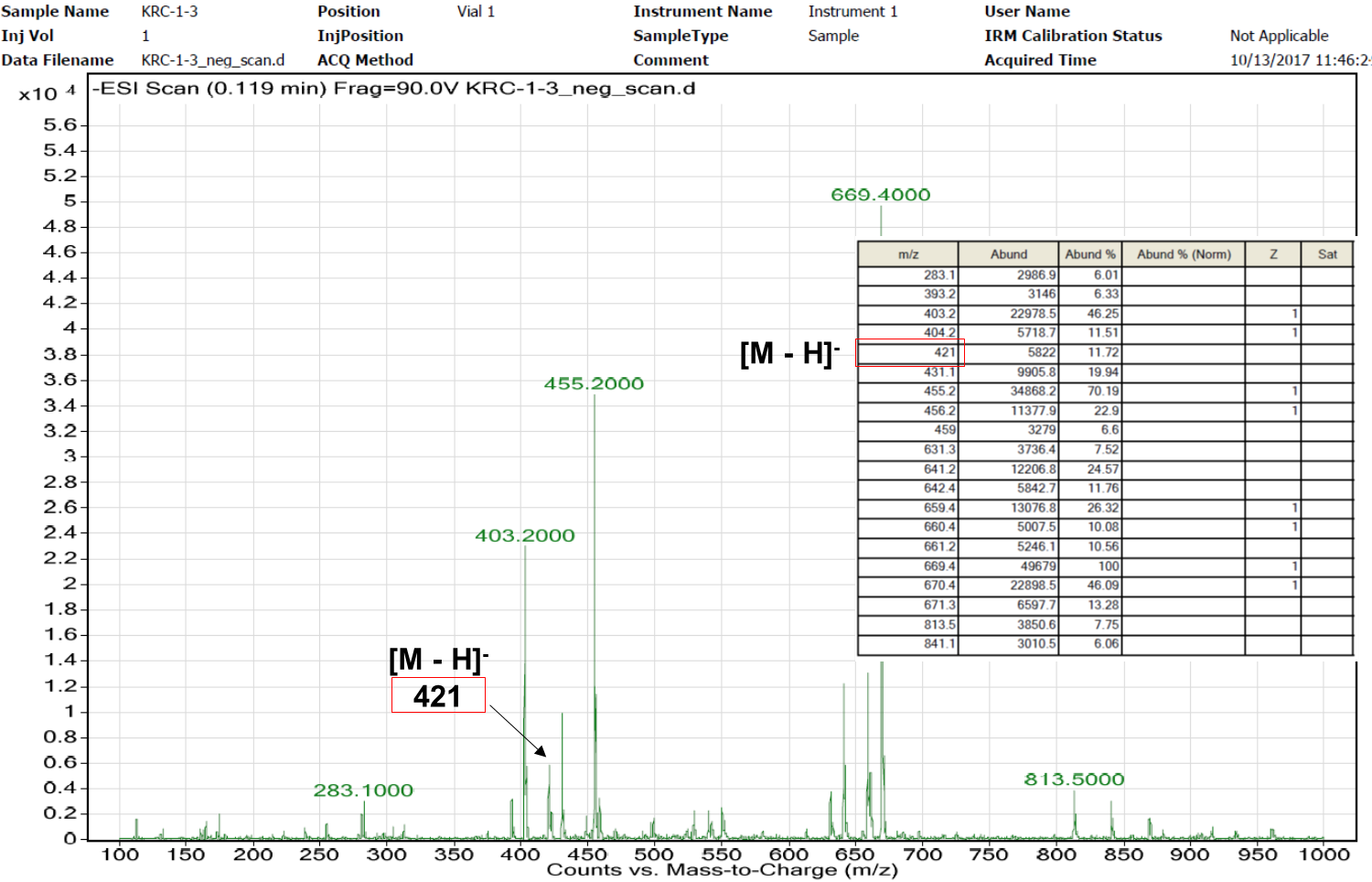
**

**Figure S1.** ESIMS spectrum of **1**.

**Figure S2.** ^1^H NMR (400 MHz, chloroform-*d*) spectrum of **1**.

**Figure S3.** ^13^C NMR (100 MHz, chloroform-*d*) spectrum of **1**.

**
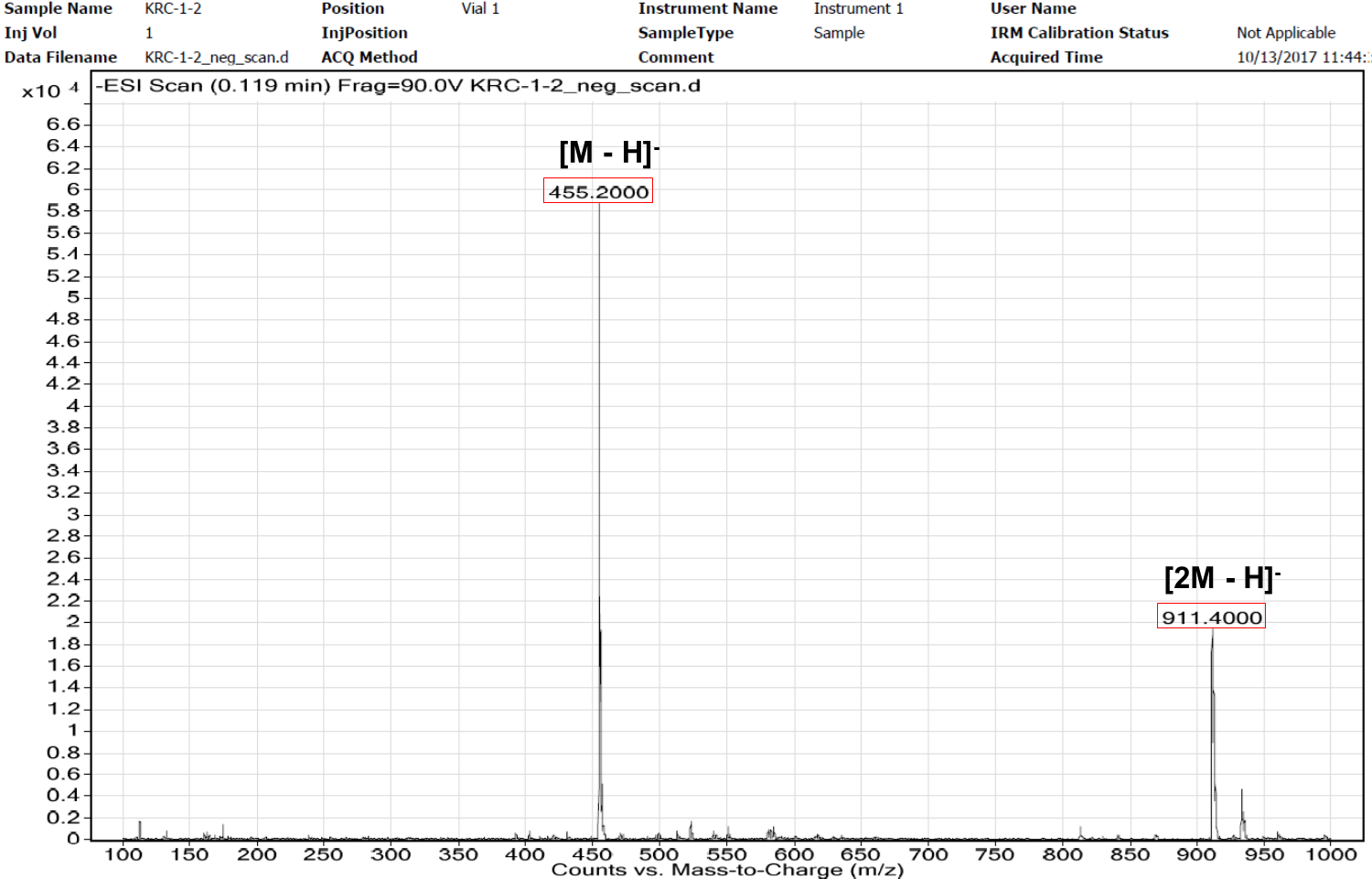
**

**Figure S4.** ESIMS spectrum of **2**.

**Figure S5.** ^1^H NMR (600 MHz, pyridine-*d*_5_) spectrum of **2**.

**Figure S6.** ^13^C NMR (150 MHz, pyridine-*d*_5_) spectrum of **2**.


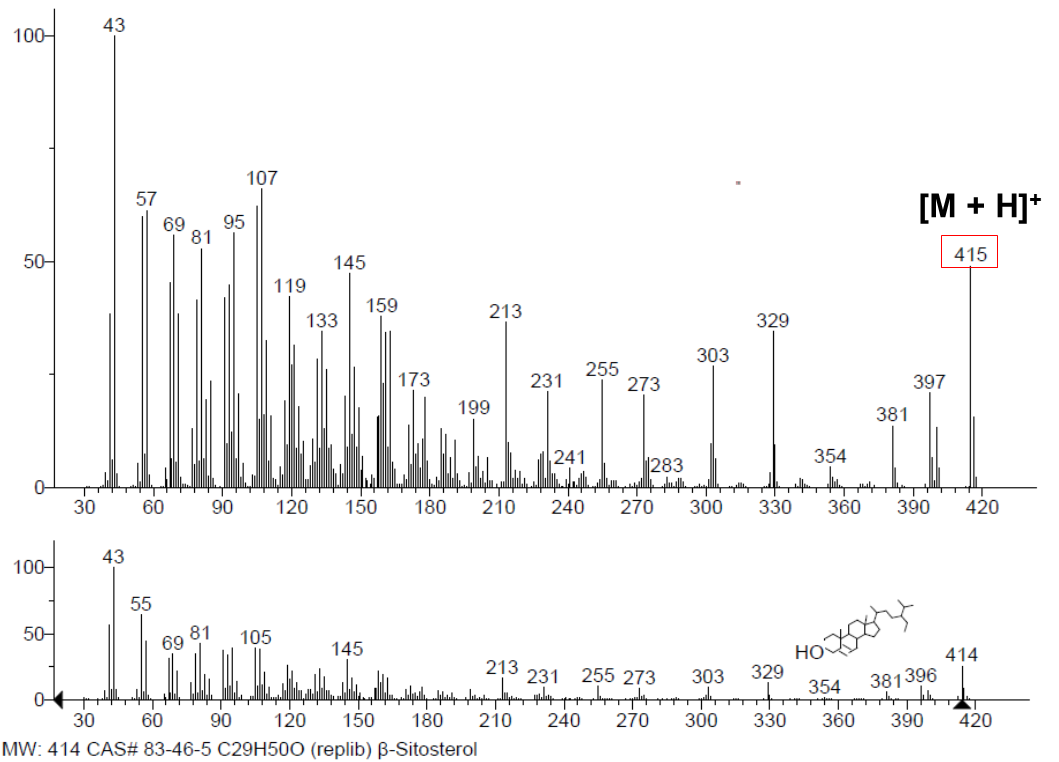


**Figure S7.** GC-MS spectrum of **3**.

**
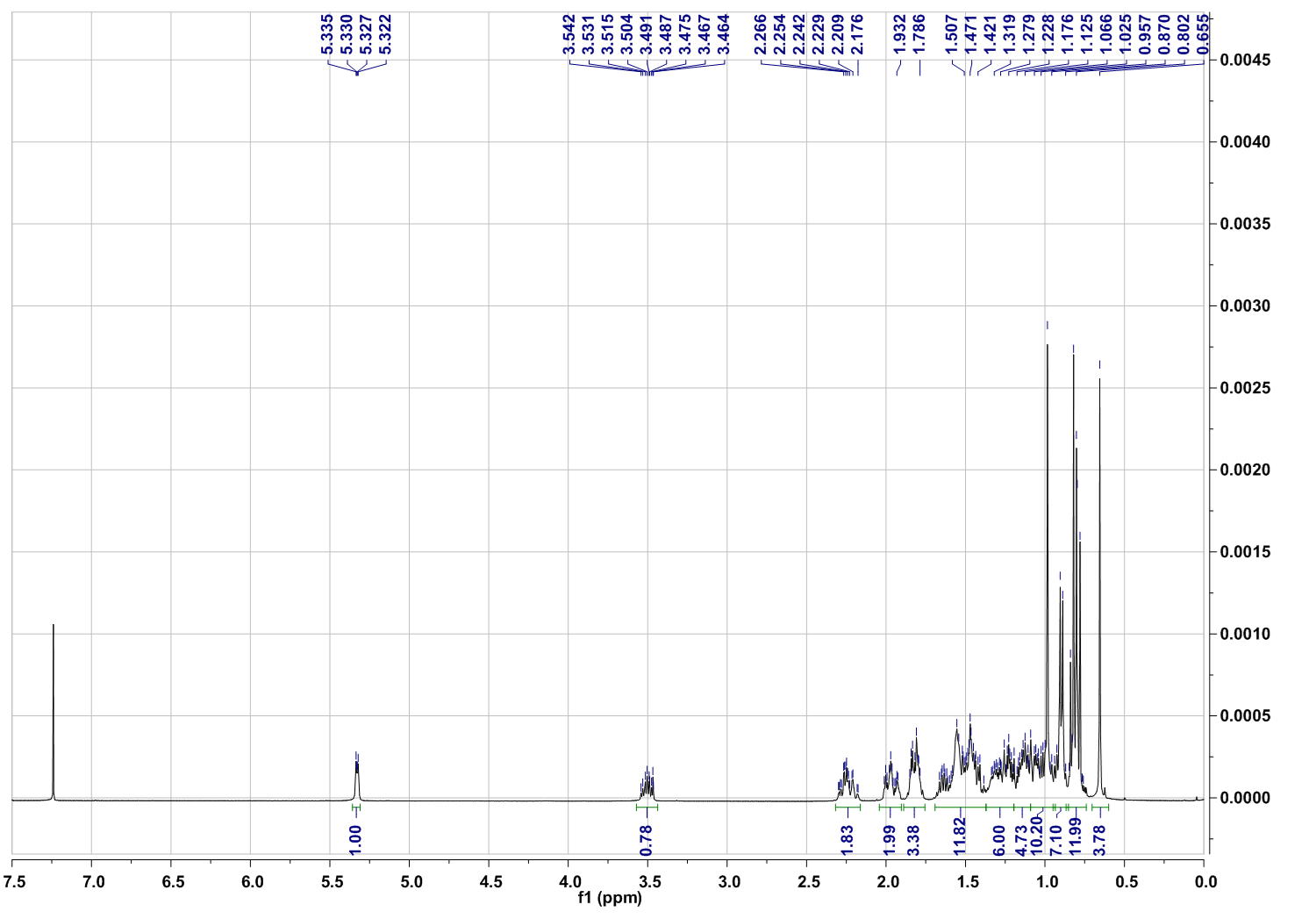
**

**Figure S8.** ^1^H NMR (400 MHz, chloroform-*d*) spectrum of **3**.

**
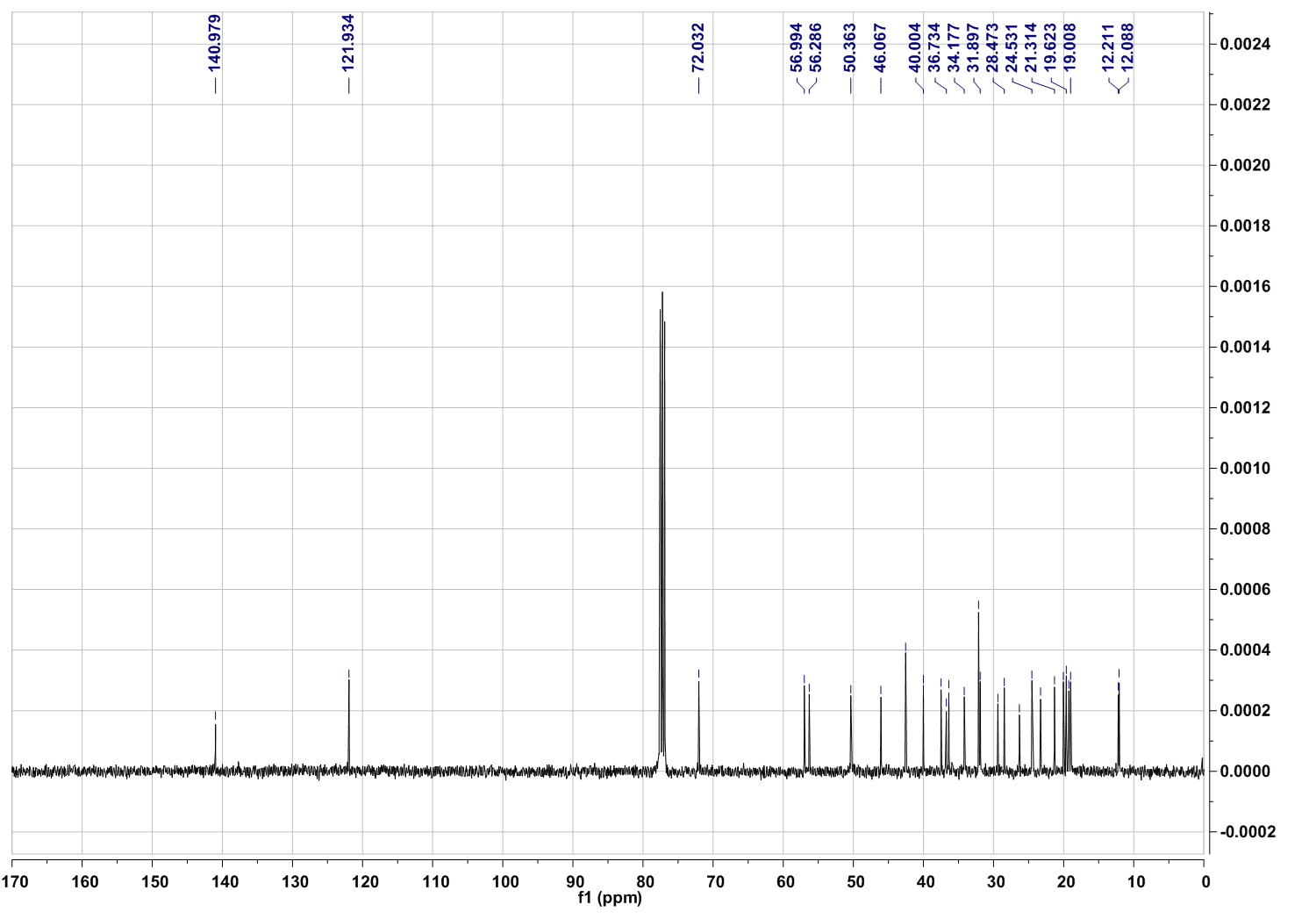
**

**Figure S9.** ^13^C NMR (100 MHz, chloroform-*d*) spectrum of **3**.

**
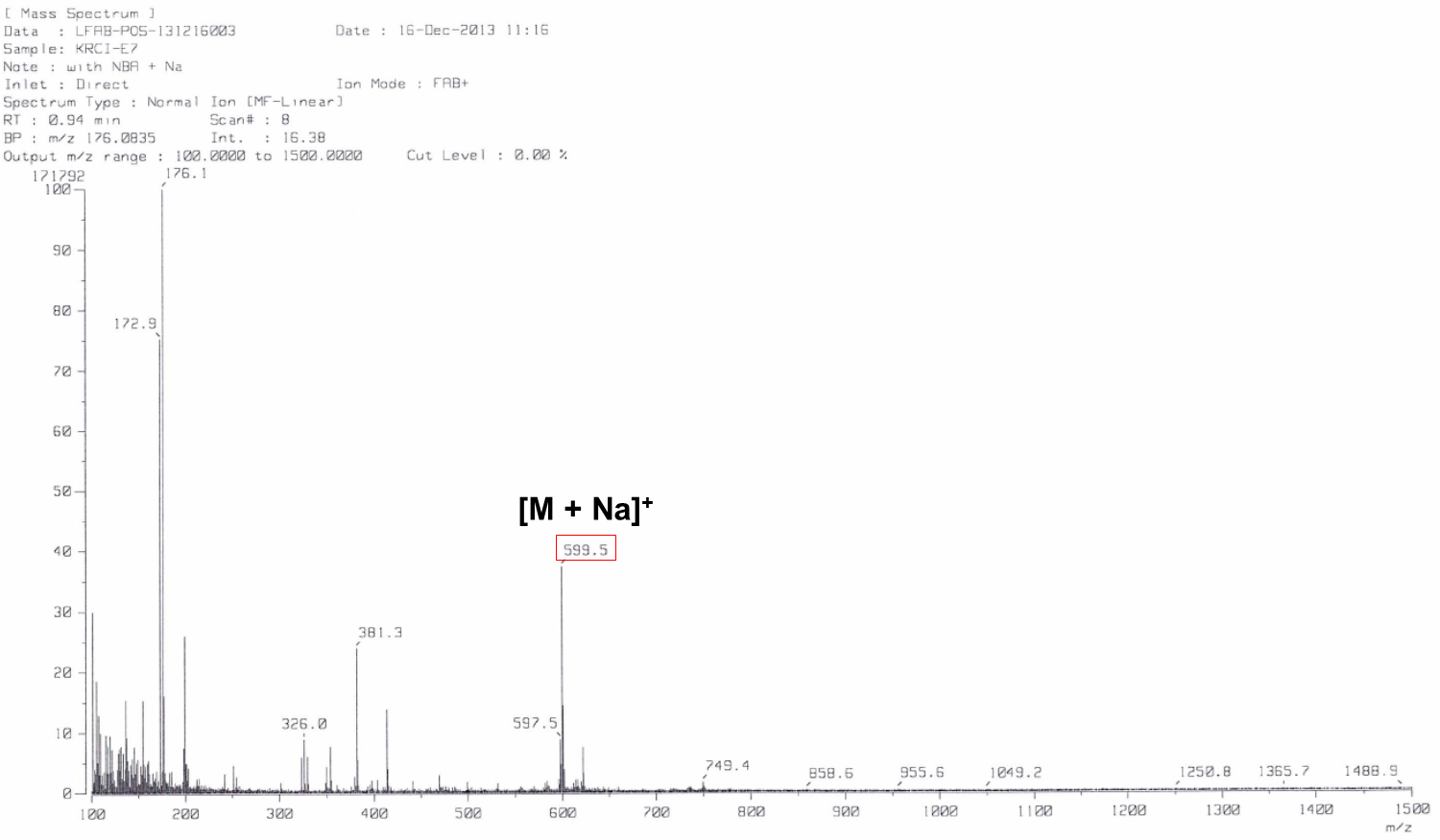
**

**Figure S10.** FABMS spectrum of **4**.

**
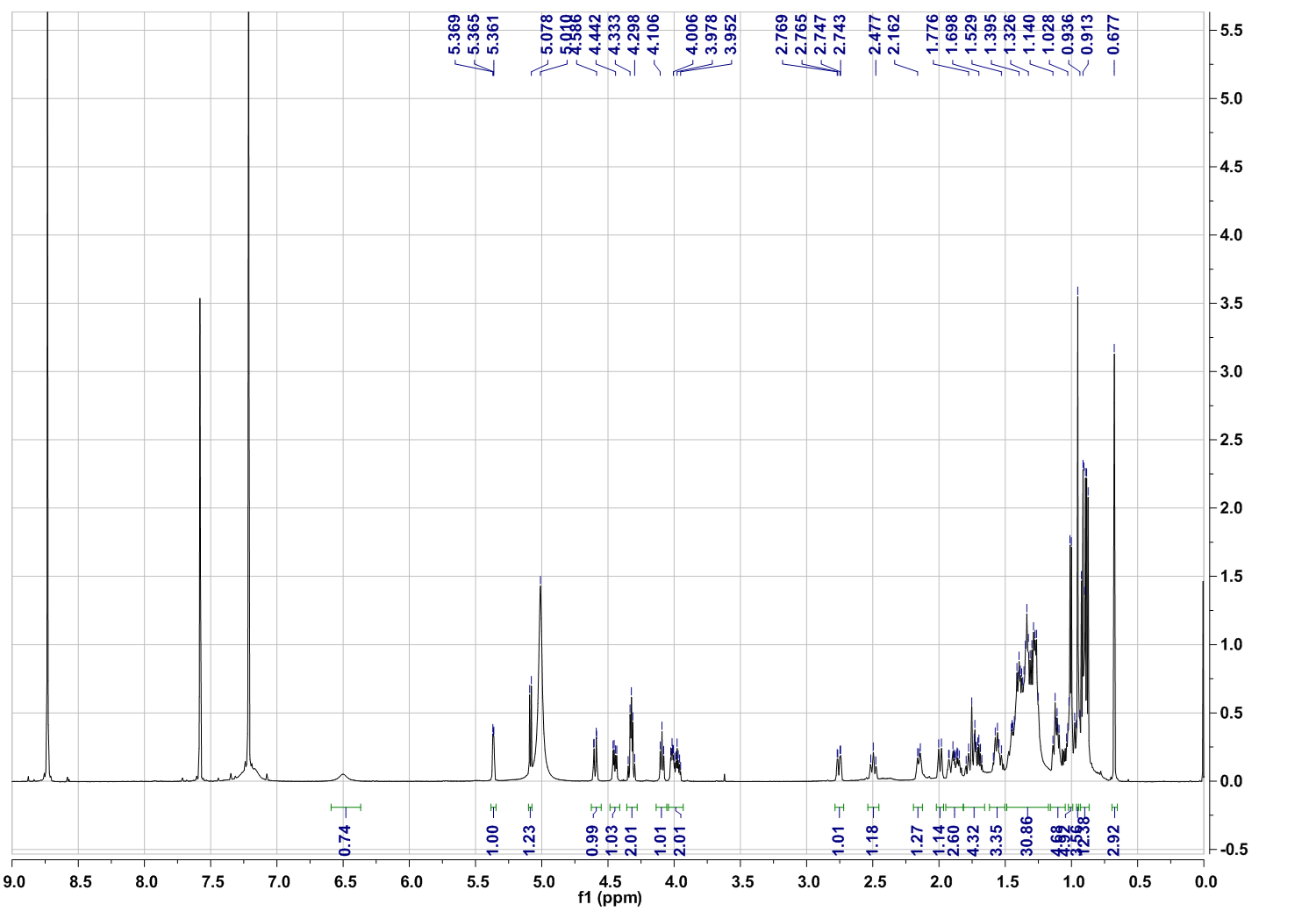
**

**Figure S11.** ^1^H NMR (600 MHz, pyridine-*d*_5_) spectrum of **4**.

**
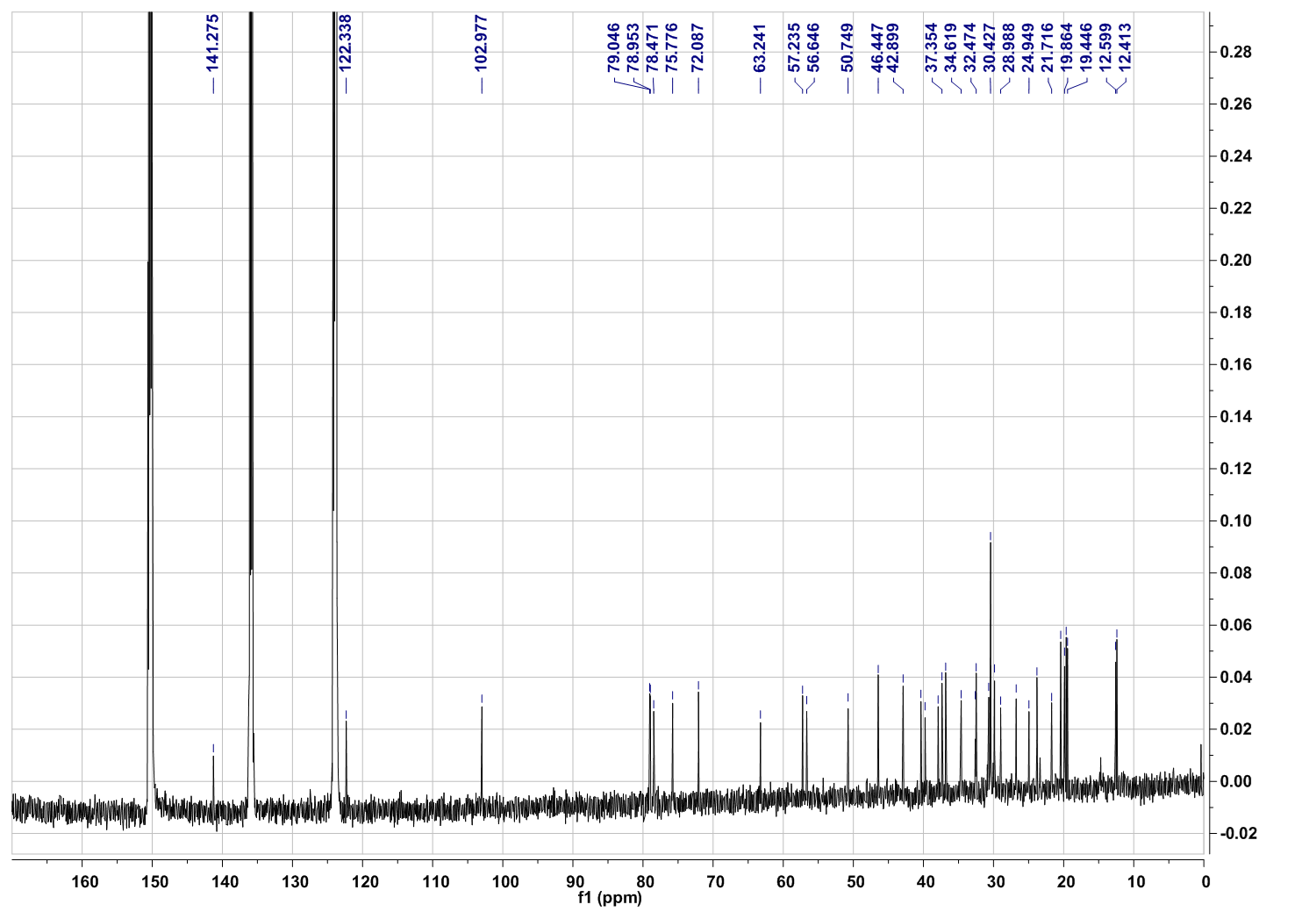
**

**Figure S12.** ^13^C NMR (150 MHz, pyridine-*d*_5_) spectrum of **4**.

**
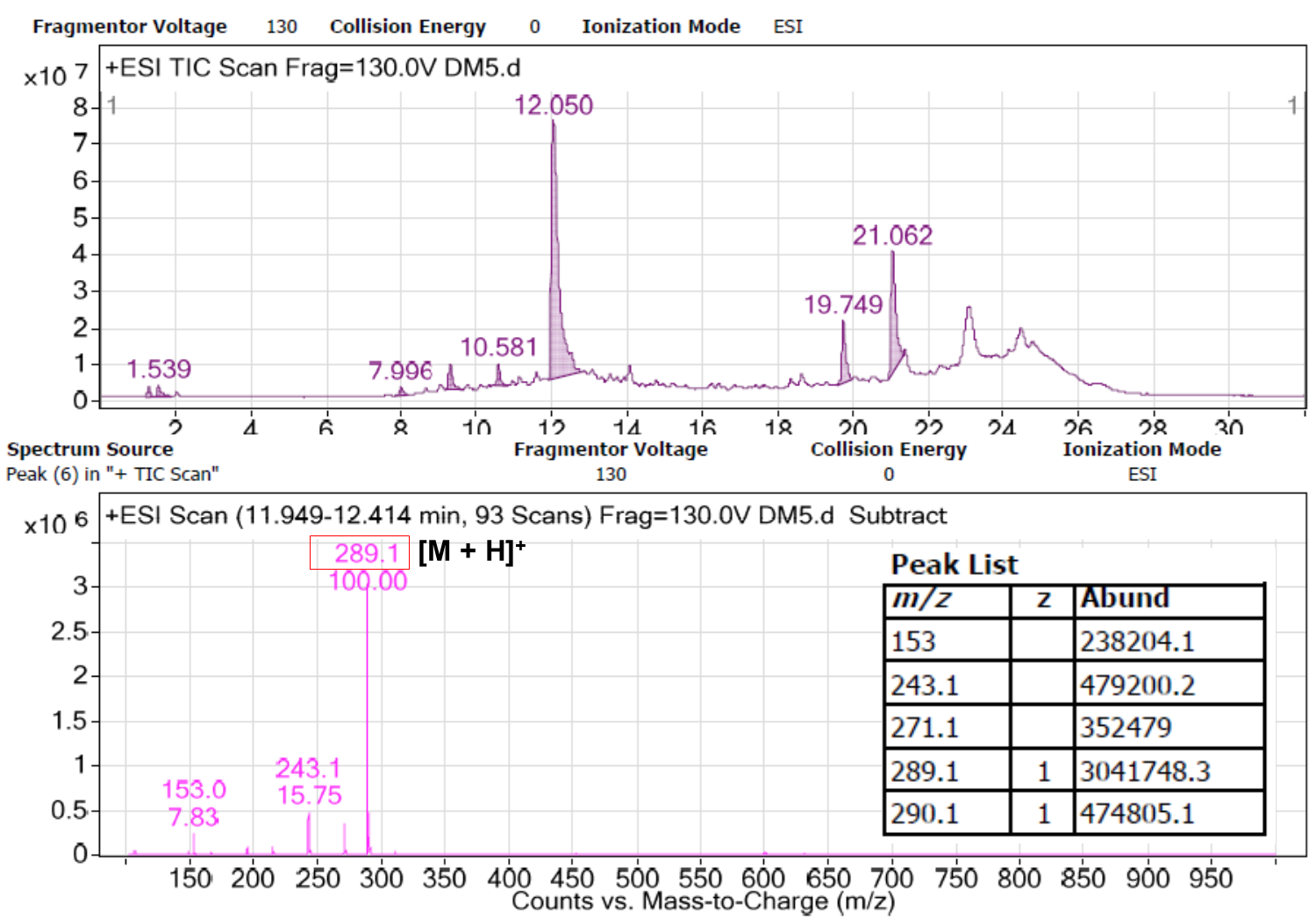
**

**Figure S13.** ESIMS spectrum of **5**.

**
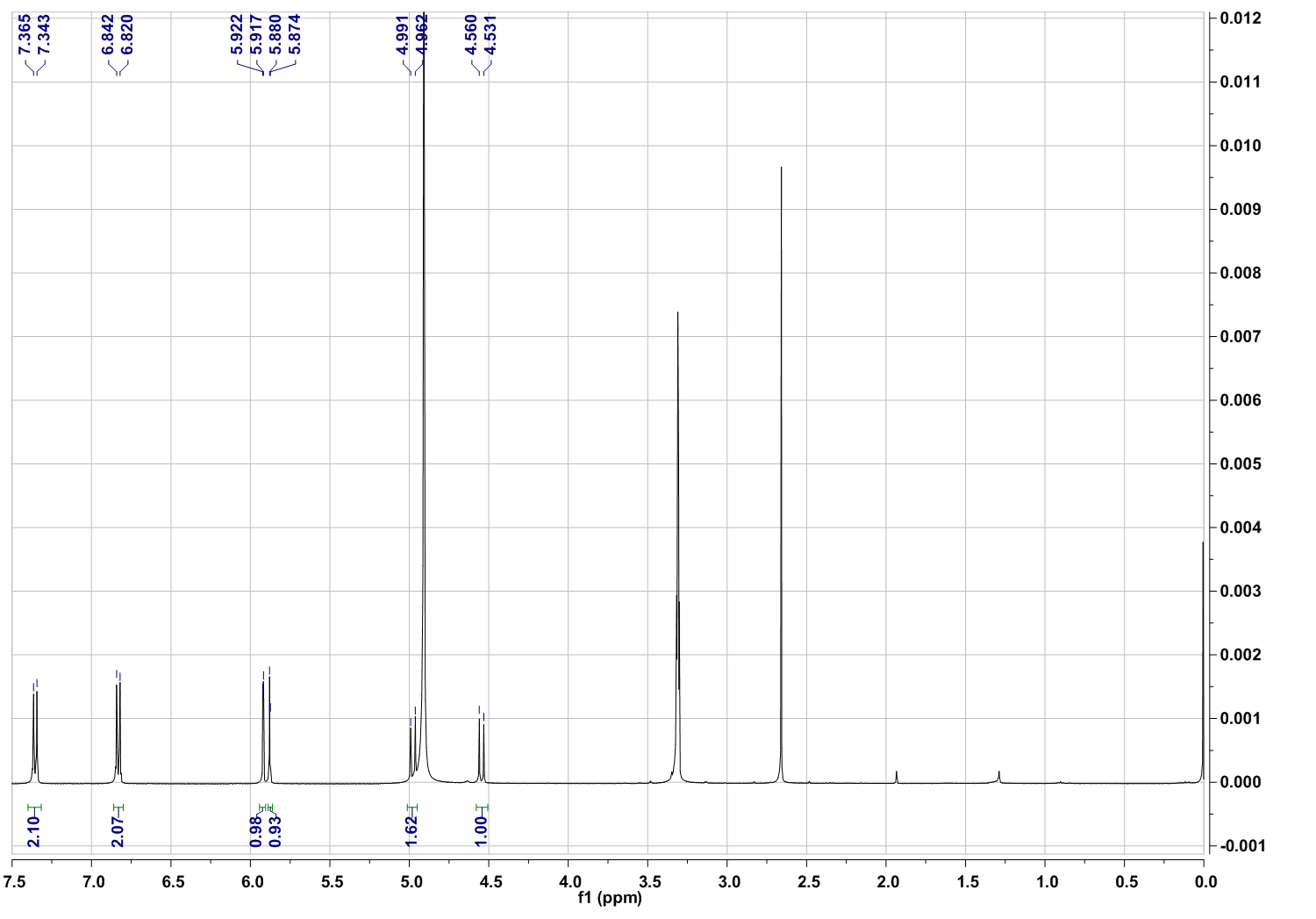
**

**Figure S14.** ^1^H NMR (400 MHz, methanol-*d*_4_) spectrum of **5**.

**Figure S15.** ^13^C NMR (100 MHz, methanol-*d*_4_) spectrum of **5**.

**
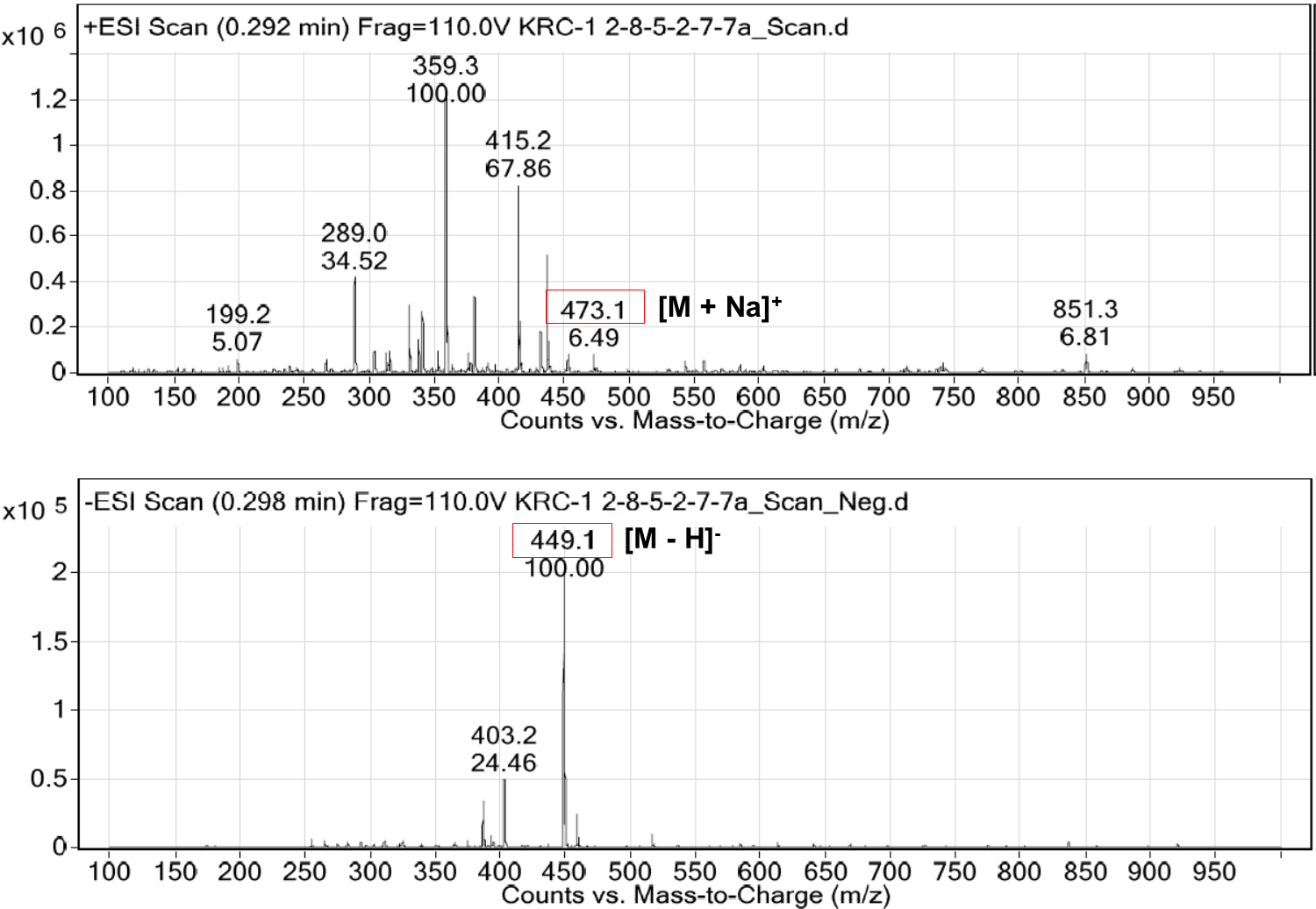
**

**Figure S15.** ESIMS spectrum of **6**.

**Figure S17.** ^1^H NMR (400 MHz, methanol-*d*_4_) spectrum of **6**.

**Figure S18.** ^13^C NMR (100 MHz, methanol-*d*_4_) spectrum of **6_._**

**
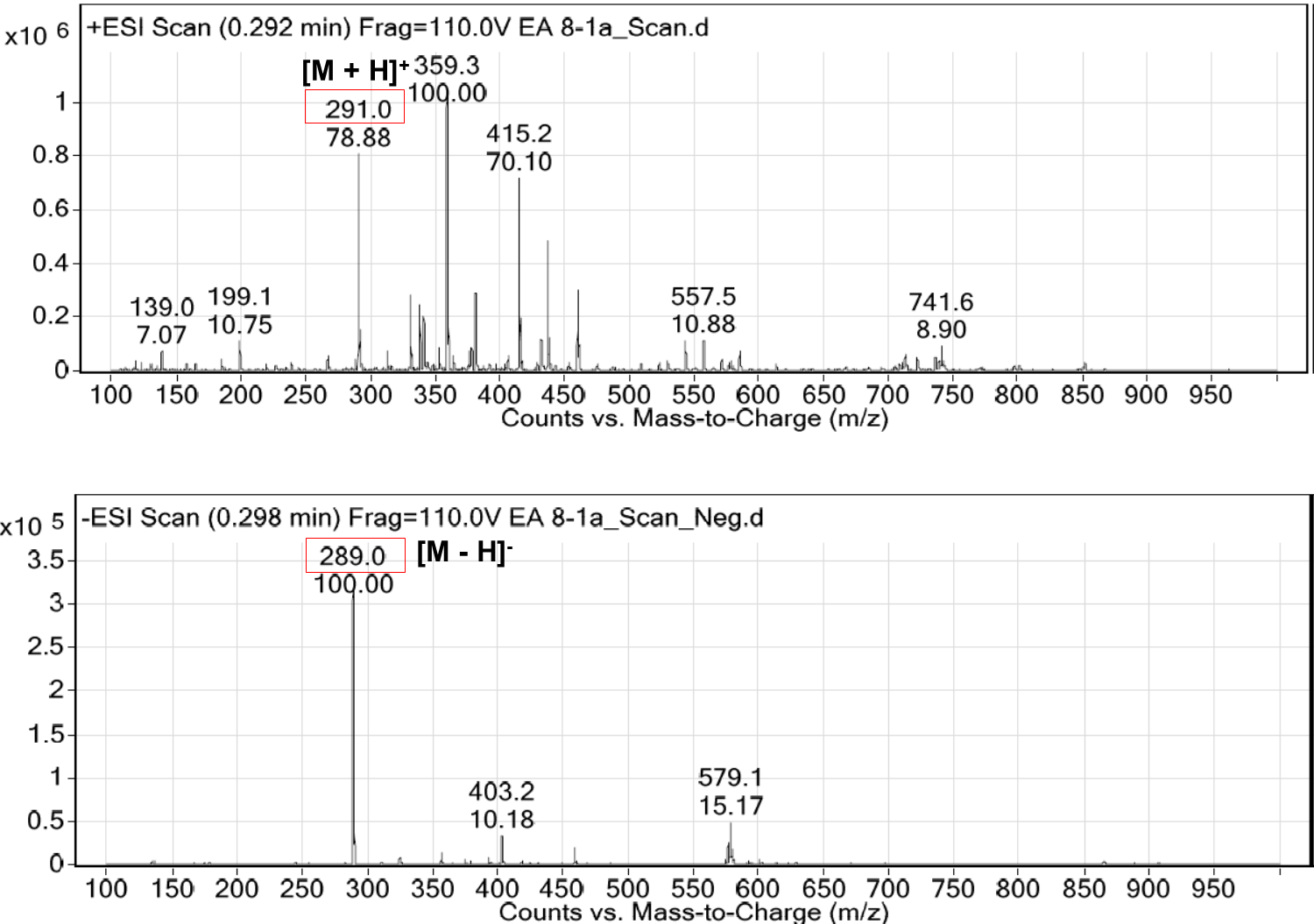
**

**Figure S19.** ESIMS spectrum of **7**.

**
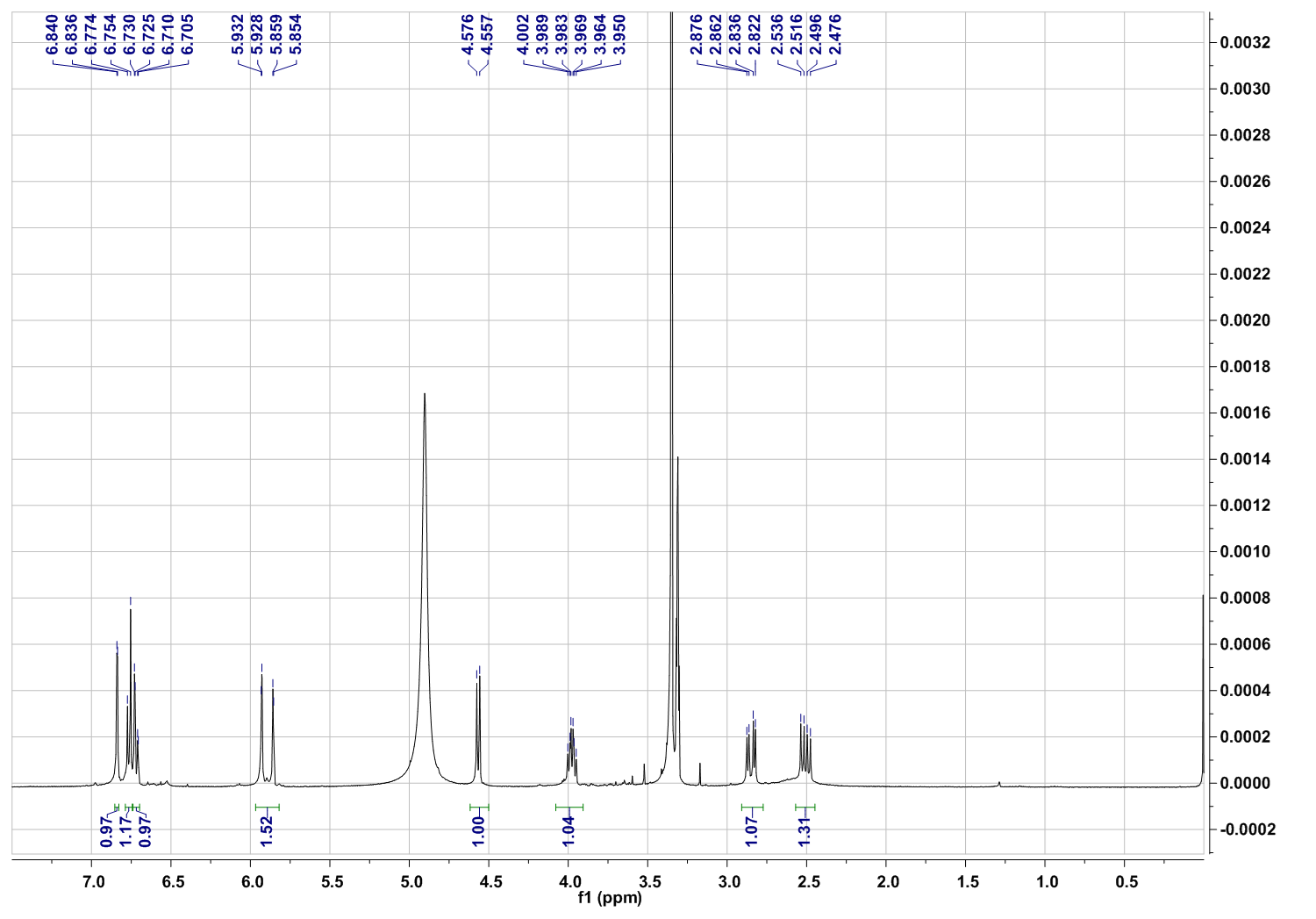
**

**Figure S20.** ^1^H NMR (400 MHz, methanol-*d*_4_) spectrum of **7**.

**Figure S20.** ^13^C NMR (100 MHz, methanol-*d*_4_) spectrum of **7**.

**
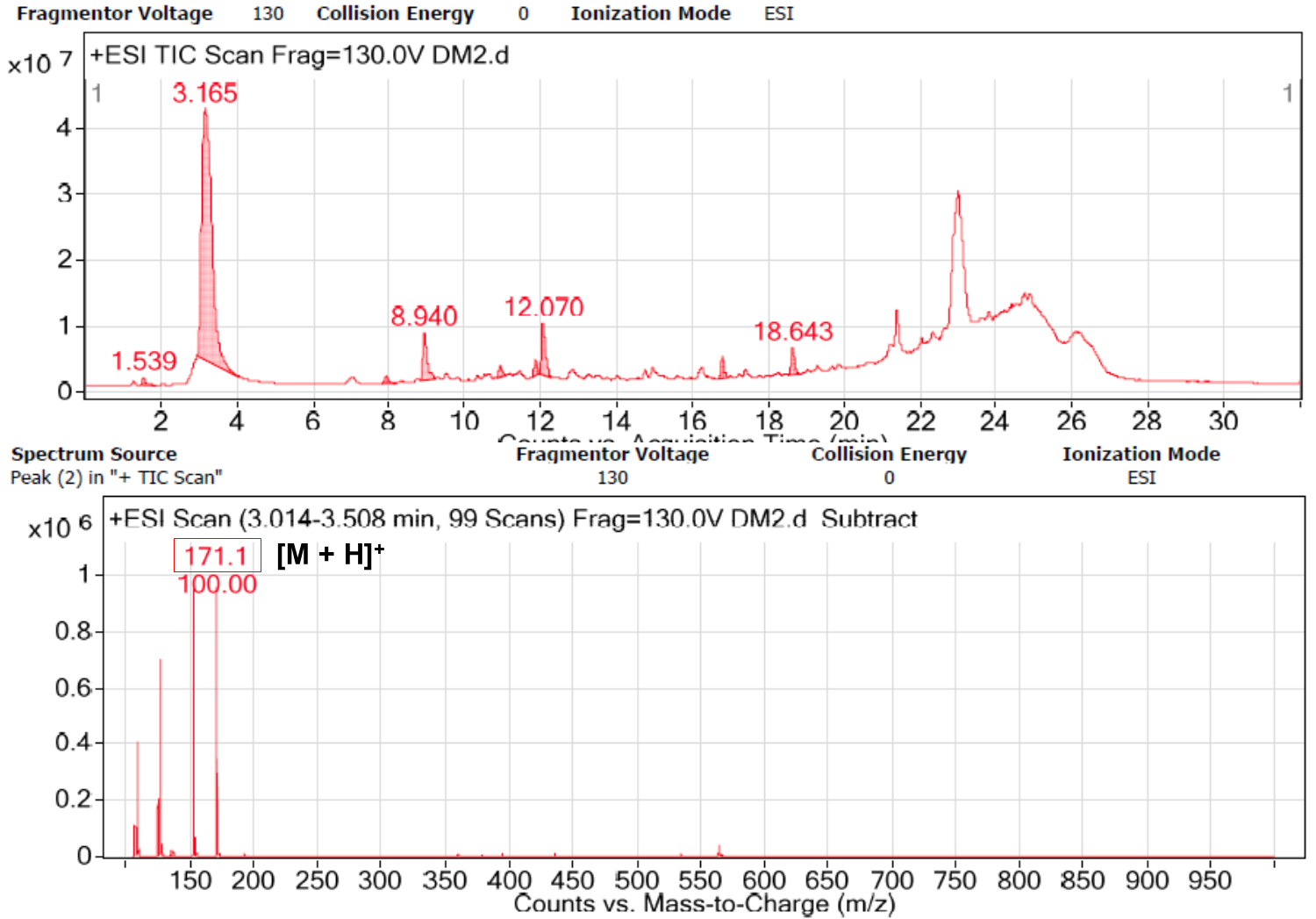
**

**Figure S22.** ESIMS spectrum of **8**.

**Figure S23.** ^1^H NMR (400 MHz, methanol-*d*_4_) spectrum of **8**.

**Figure S24.** ^13^C NMR (100 MHz, methanol-*d*_4_) spectrum of **8.**

**
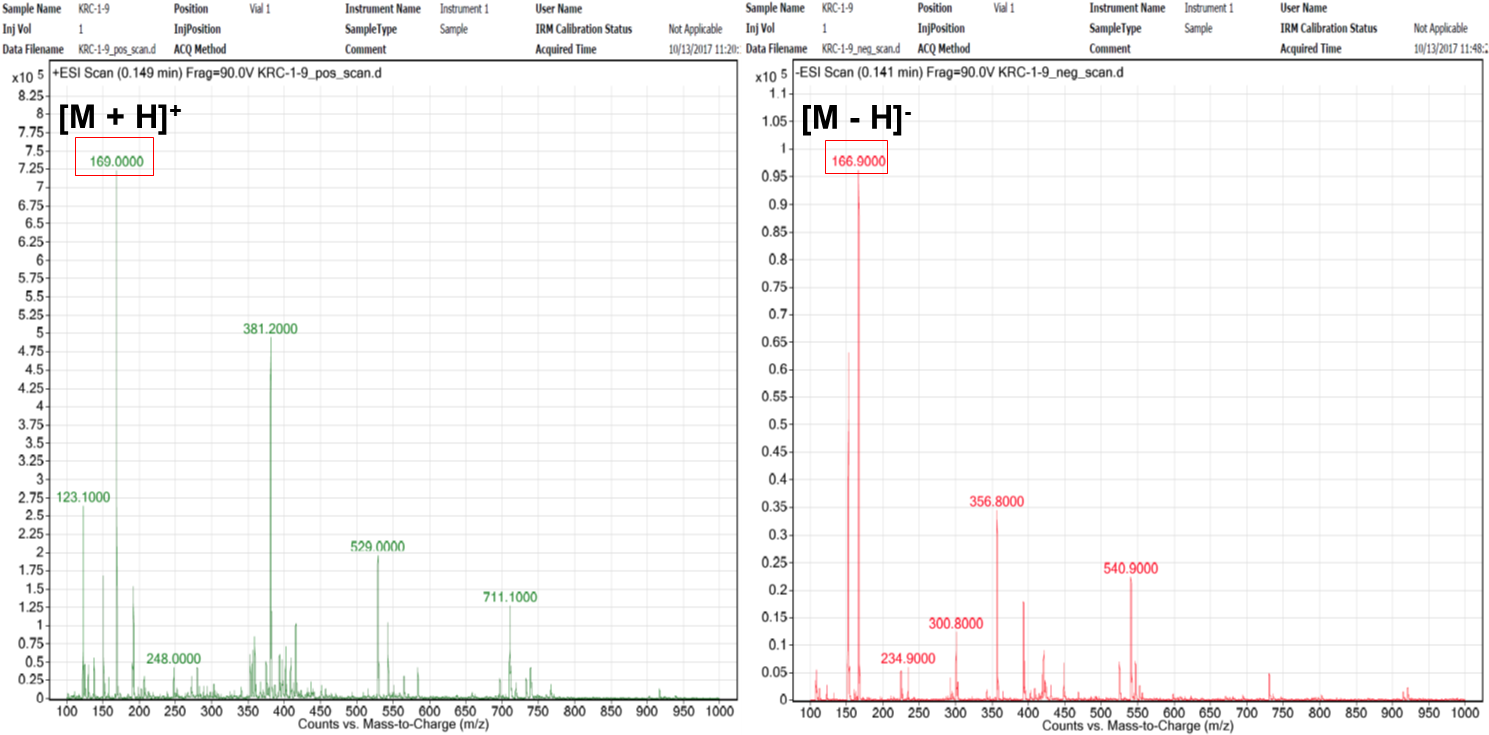
**

**Figure S25.** ESIMS spectrum of **9**.

**Figure S26.** ^1^H NMR (600 MHz, methanol-*d*_4_) spectrum of **9**.

**Figure S27.** ^13^C NMR (150 MHz, methanol-*d*_4_) spectrum of **9**.

**
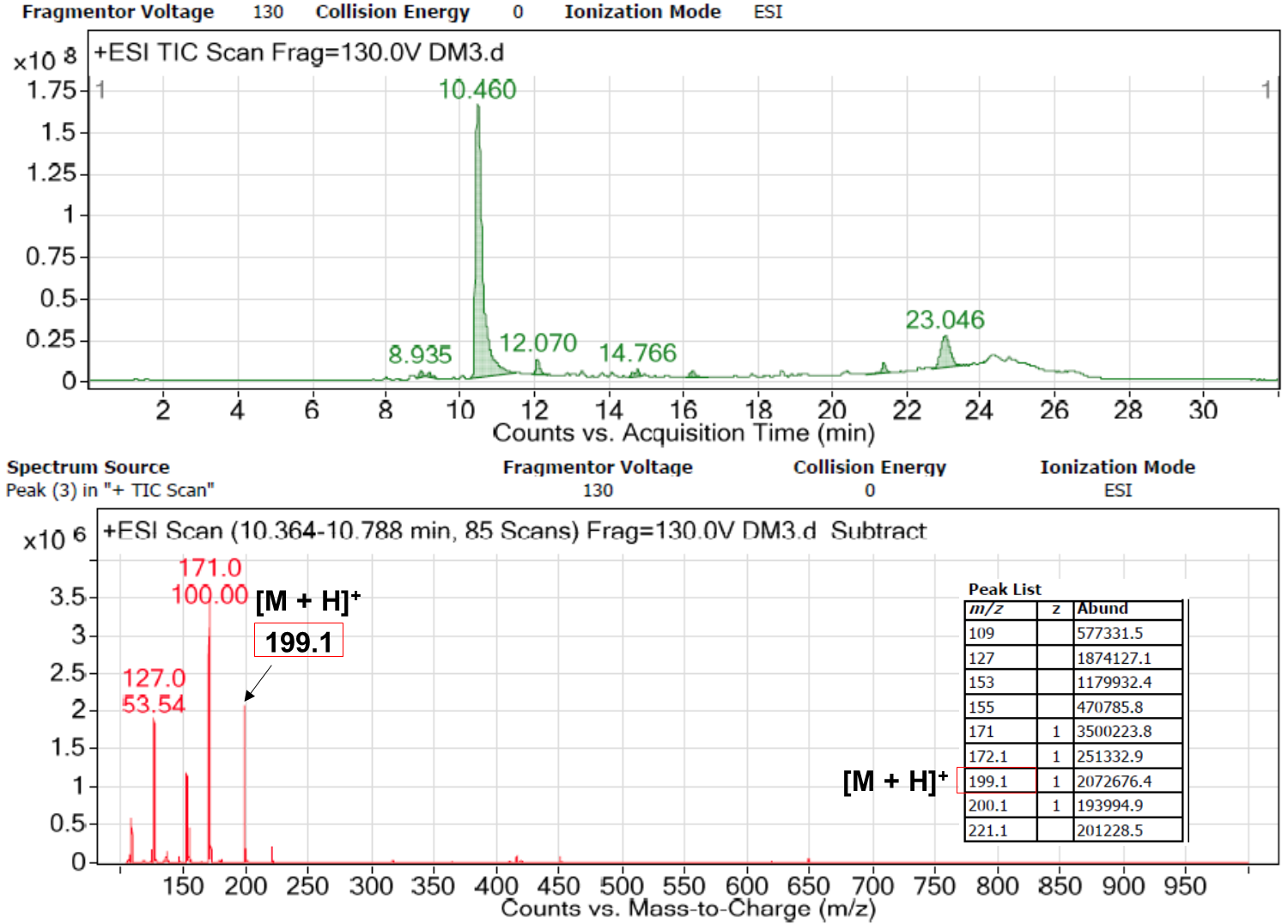
**

**Figure S28.** ESIMS spectrum of **10**.

**Figure S29.** ^1^H NMR (400 MHz, methanol-*d*_4_) spectrum of **10**.

**Figure S30.** ^13^C NMR (100 MHz, methanol-*d*_4_) spectrum of **10**.

**
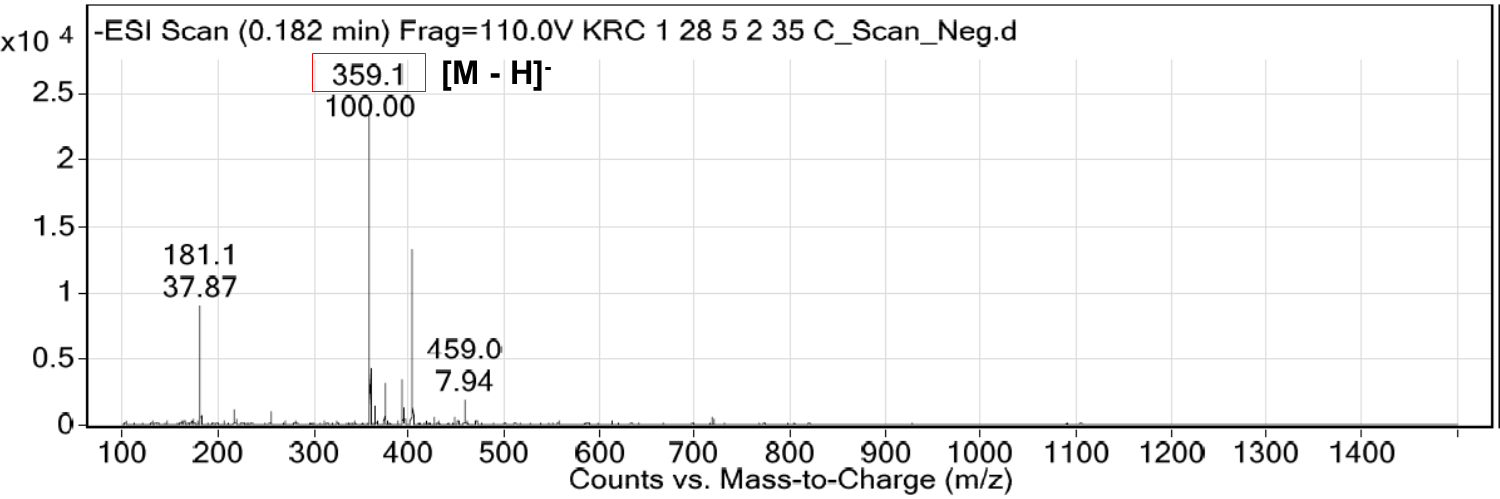
**

**Figure S31.** ESIMS spectrum of **11**.

**Figure S32.** ^1^H NMR (400 MHz, methanol-*d*_4_) spectrum of **11**.

**Figure S33.** ^13^C NMR (100 MHz, methanol-*d*_4_) spectrum of **11**.

**
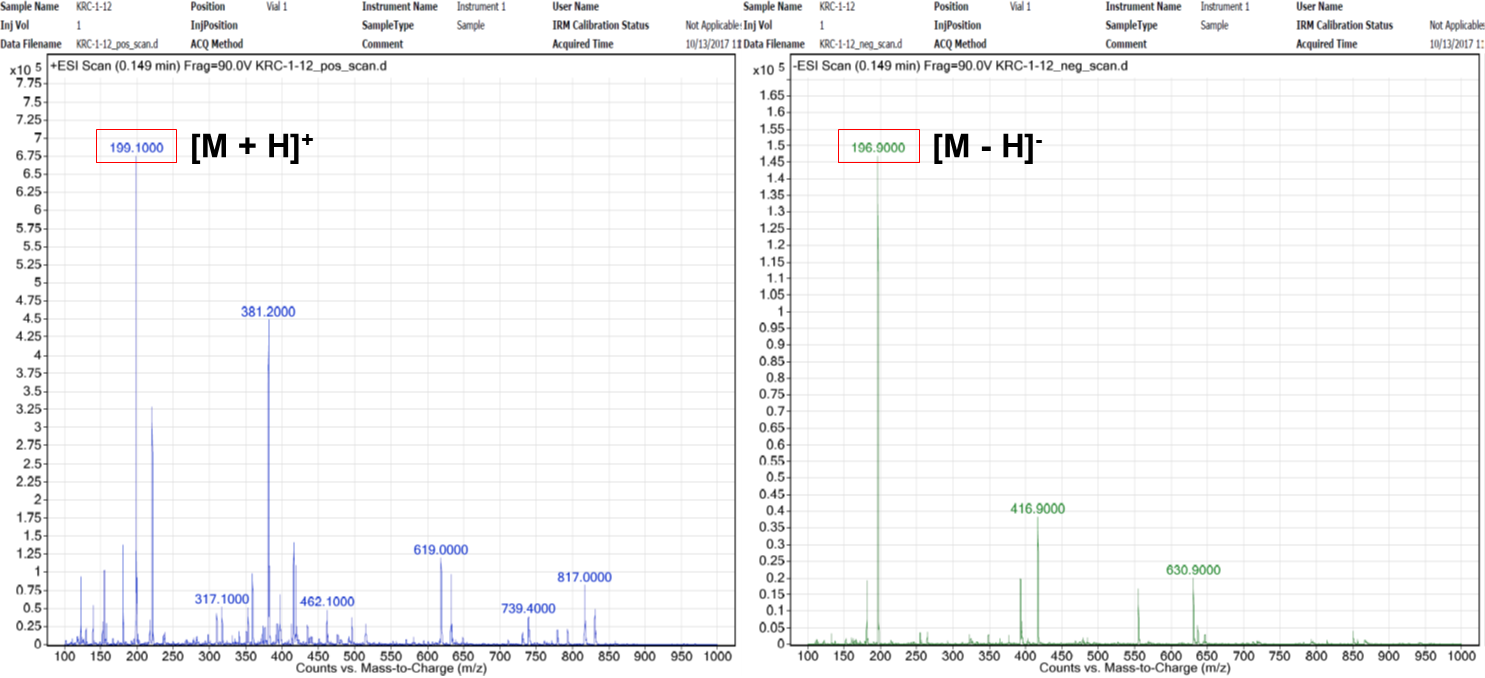
**

**Figure S34.** ESIMS spectrum of **12**.

**Figure S35.** ^1^H NMR (600 MHz, methanol-*d*_4_) spectrum of **12**.

**Figure S36.** ^13^C NMR (150 MHz, methanol-*d*_4_) spectrum of **12**.

**
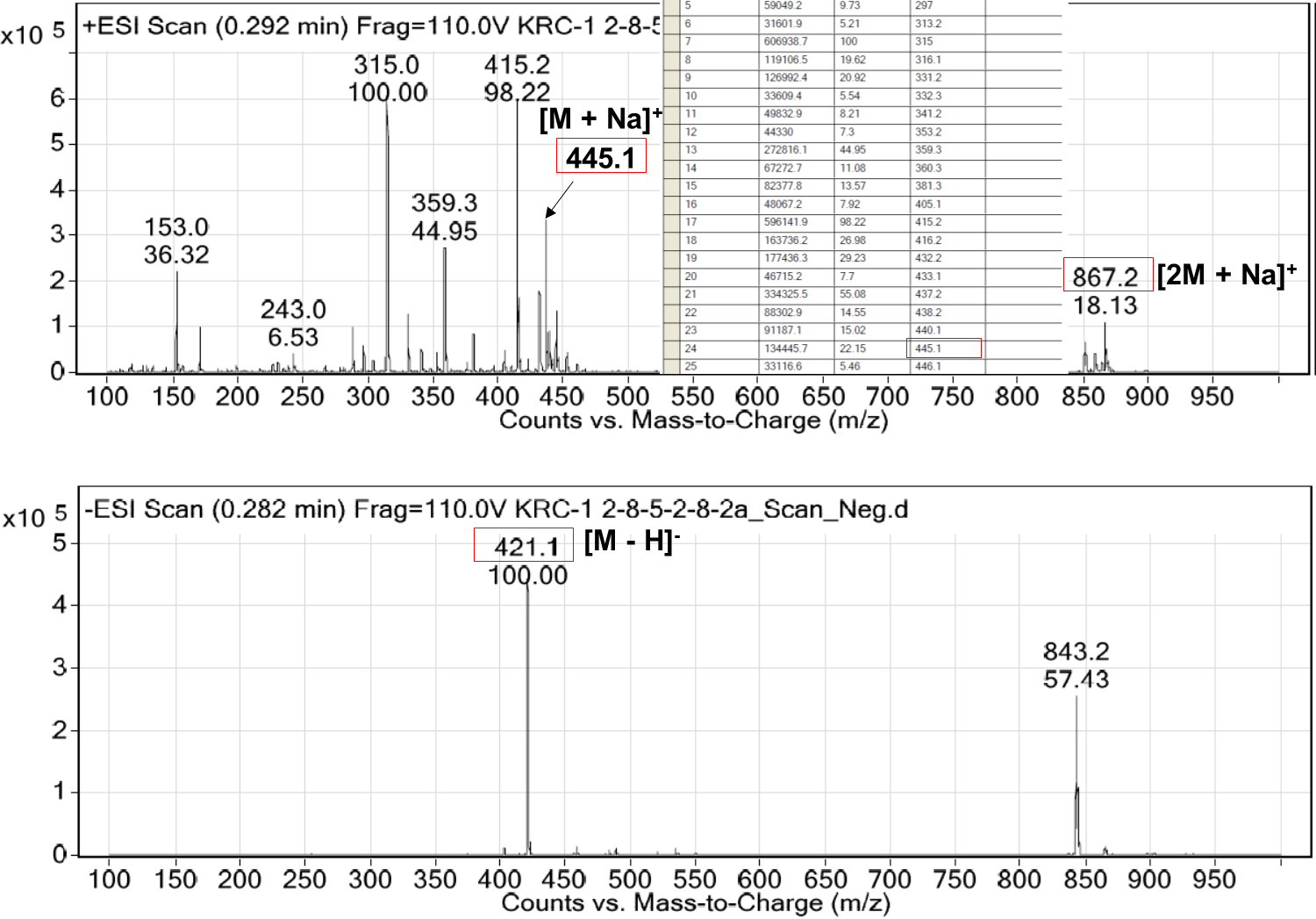
**

**Figure S37.** ESIMS spectrum of **13**.

**Figure S38.** ^1^H NMR (400 MHz, methanol-*d*_4_) spectrum of **13**.

**Figure S39.** ^13^C NMR (100 MHz, methanol-*d*_4_) spectrum of **13**.

**
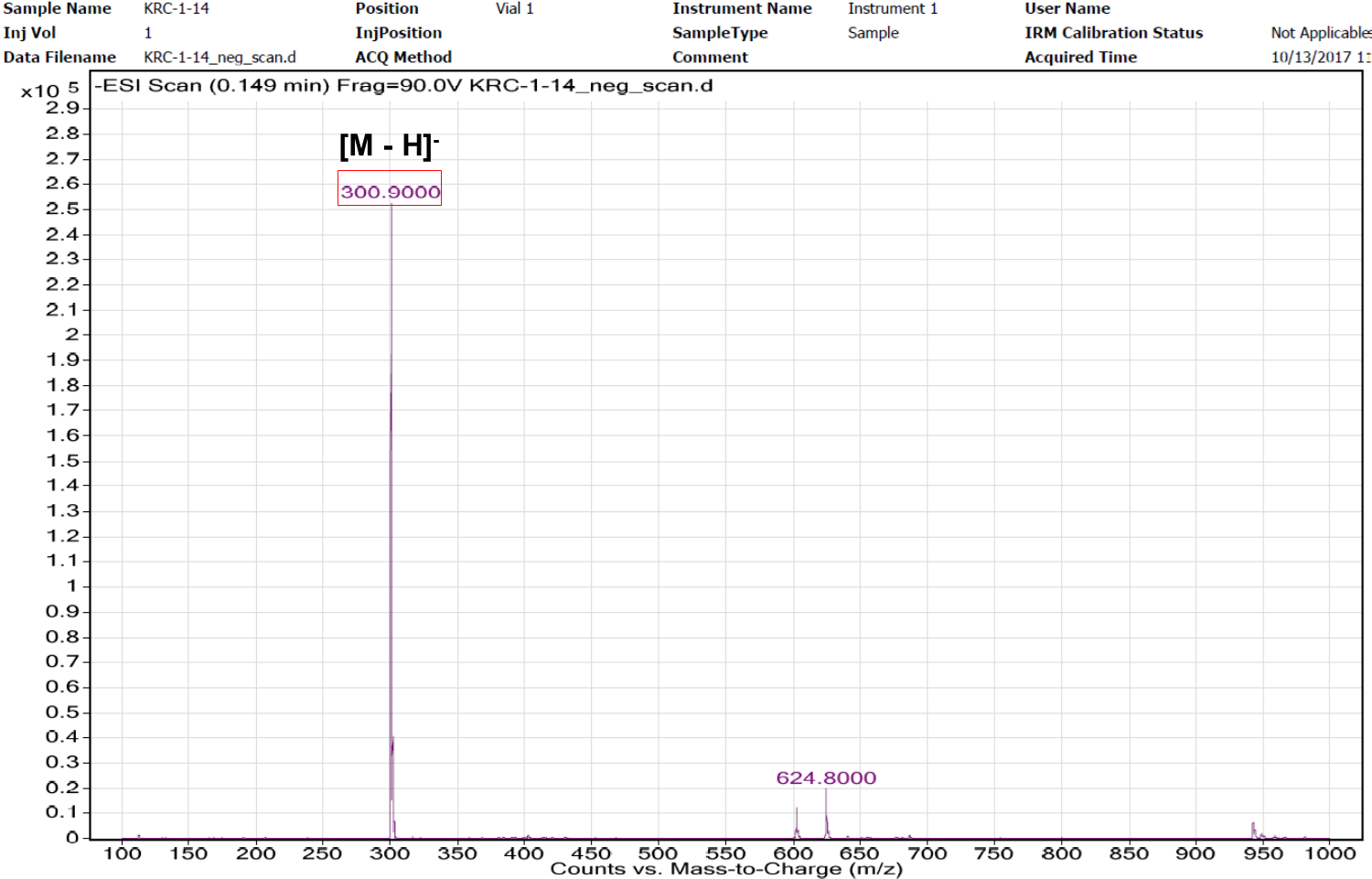
**

**Figure S40.** ESIMS spectrum of **14**.

**Figure S41.** ^1^H NMR (600 MHz, DMSO-*d*_6_) spectrum of **14**.

**Figure S42.** ^13^C NMR (100 MHz, DMSO-*d*_6_) spectrum of **14**.

**
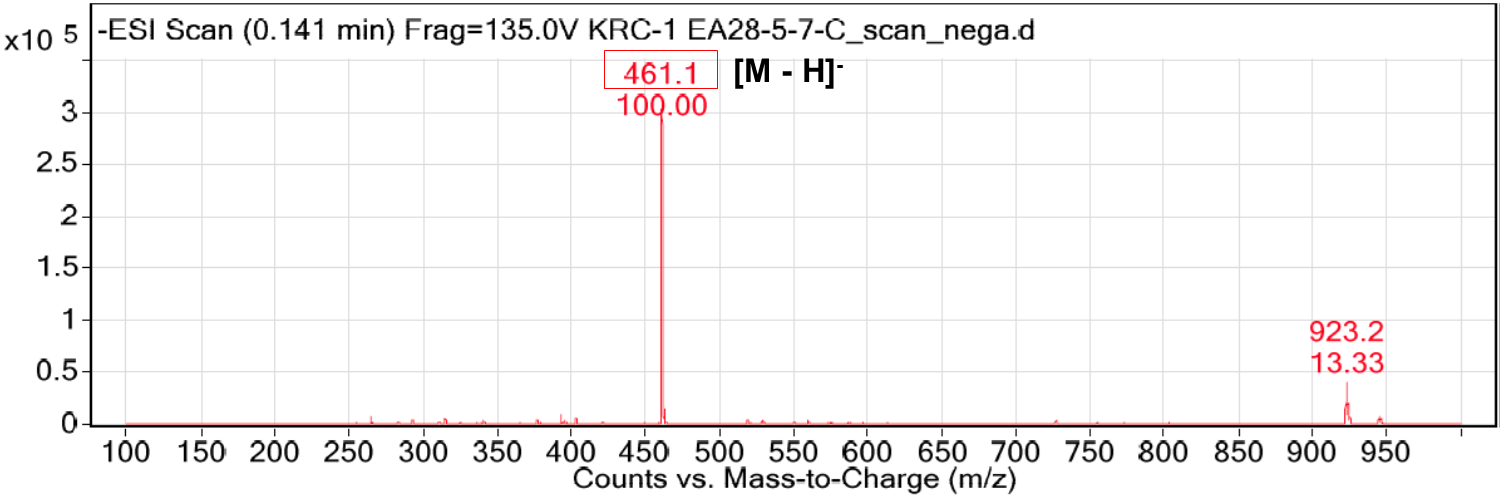
**

**Figure S43.** ESIMS spectrum of **15**

**Figure S44.** ^1^H NMR (600 MHz, DMSO-*d*_6_) spectrum of **15**.

**Figure S45.** ^13^C NMR (150 MHz, DMSO-*d*_6_) spectrum of **15**.

**
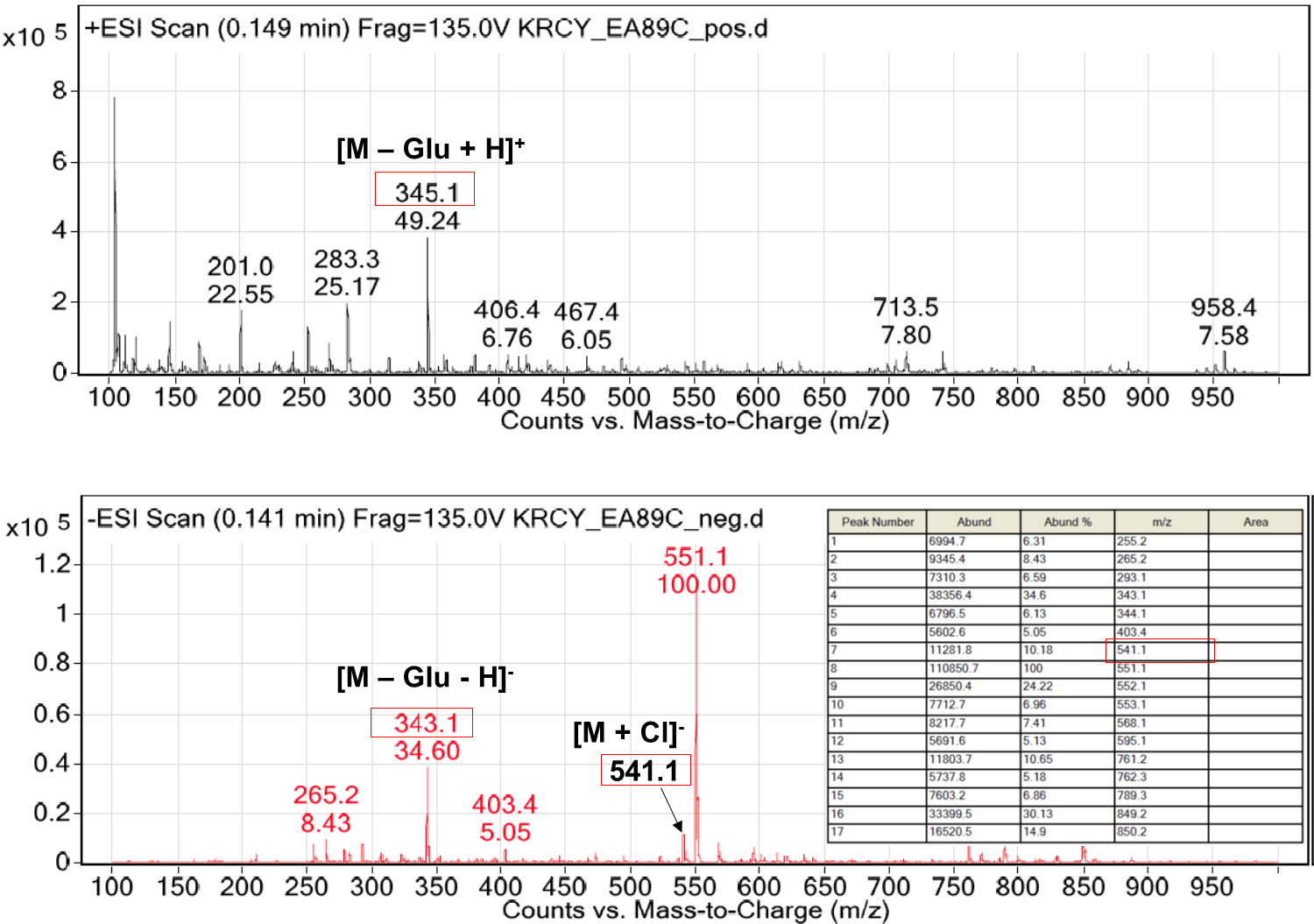
**

**Figure S46.** ESIMS spectrum of **16**.

**Figure S47.** ^1^H NMR (600 MHz, DMSO-*d*_6_) spectrum of **16**.

**Figure S48.** ^13^C NMR (150 MHz, DMSO-*d*_6_) spectrum of **16**.

**
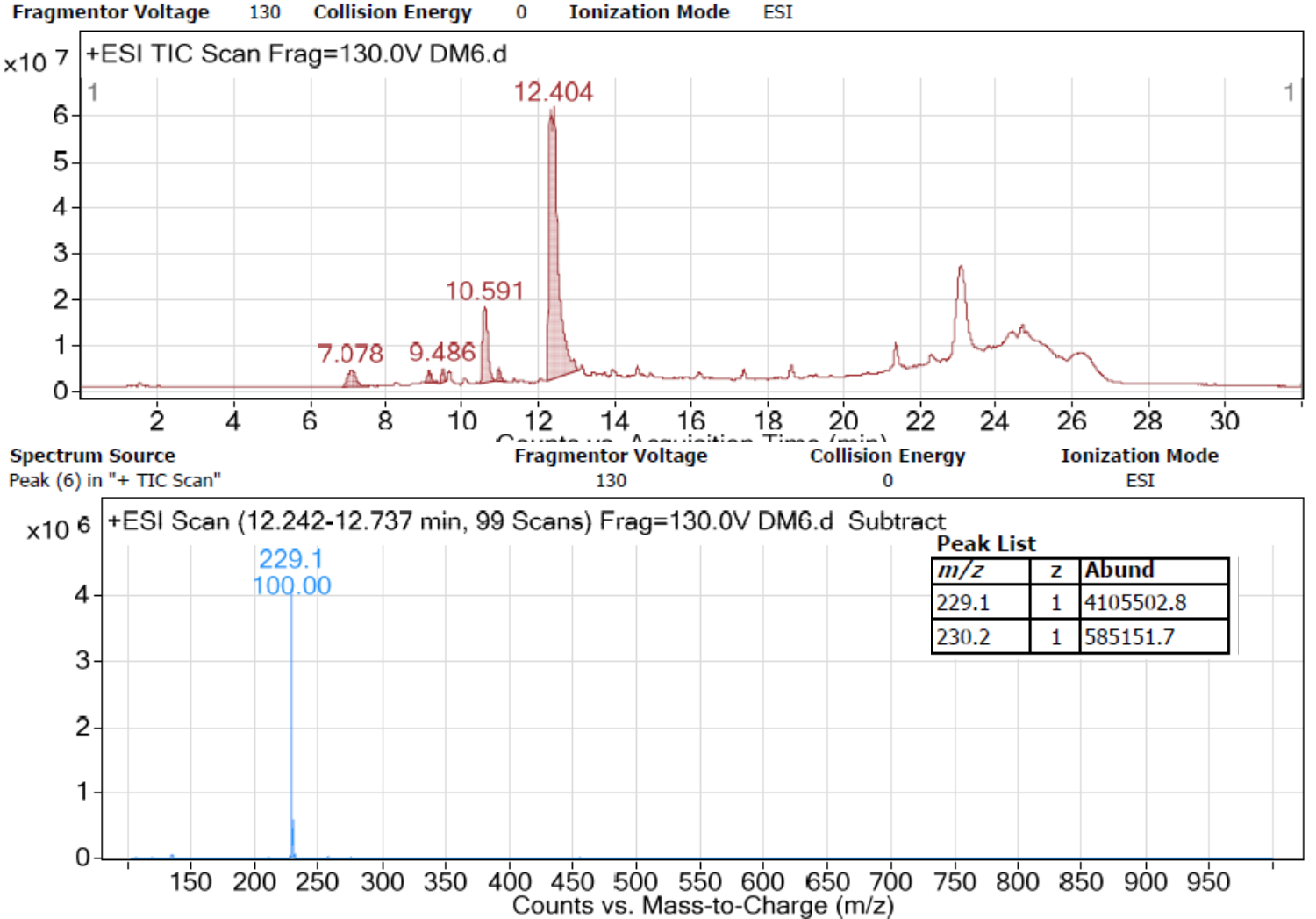
**

**Figure S49.** ESIMS spectrum of **17**.

**Figure S50.** ^1^H NMR (400 MHz, methanol-*d*_4_) spectrum of **17**.

**Figure S51.** ^13^C NMR (100 MHz, methanol-*d*_4_) spectrum of **17**.

**
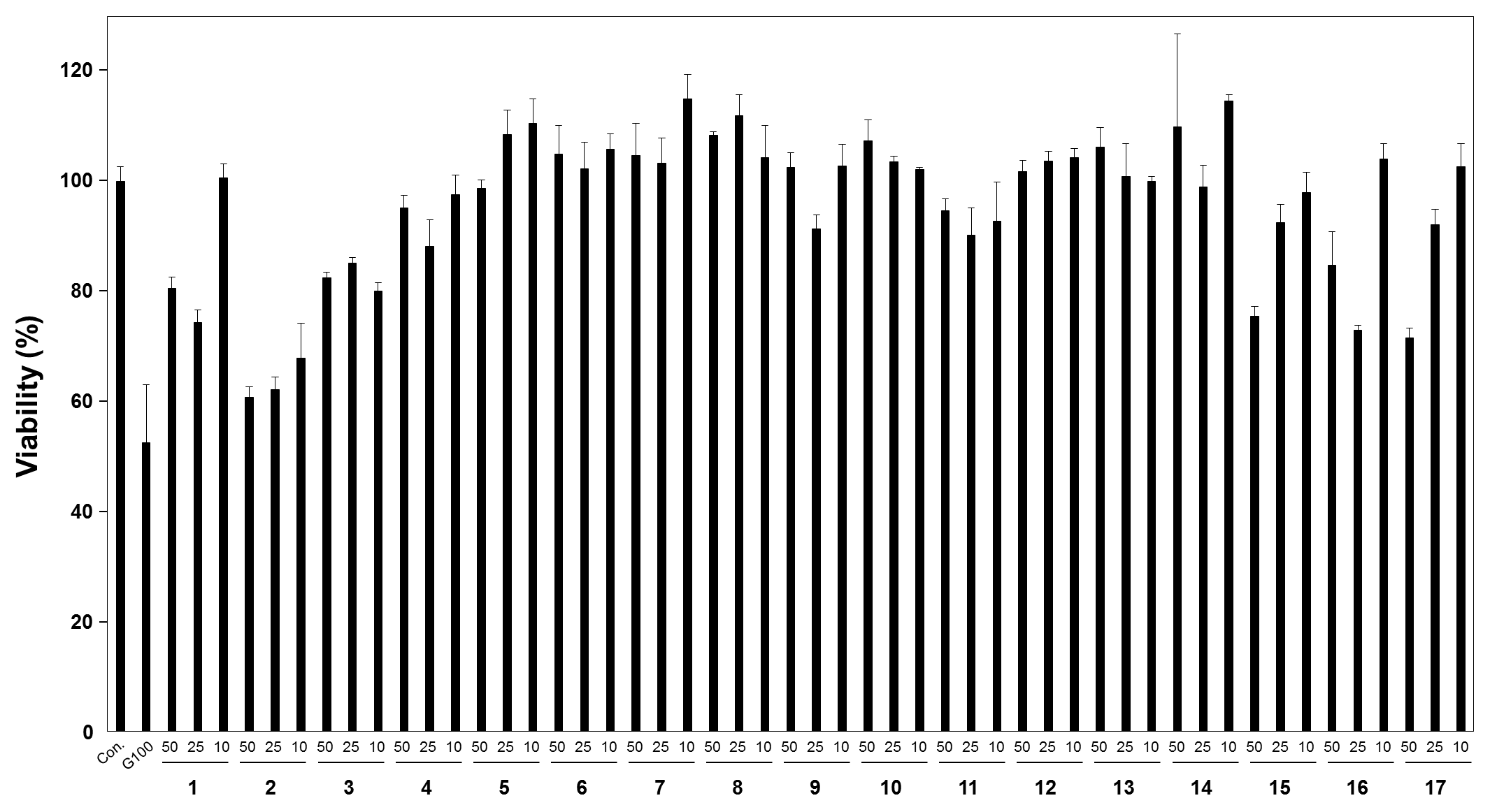
**

**Figure S52.** Cytotoxicity of compounds **1**-**17** on Hep3B-STAT3-Luc cell lines.
